# Integrative analysis of the microbiome and metabolome of the human intestinal mucosal surface reveals exquisite inter-relationships

**DOI:** 10.1186/2049-2618-1-17

**Published:** 2013-06-05

**Authors:** Ian H McHardy, Maryam Goudarzi, Maomeng Tong, Paul M Ruegger, Emma Schwager, John R Weger, Thomas G Graeber, Justin L Sonnenburg, Steve Horvath, Curtis Huttenhower, Dermot PB McGovern, Albert J Fornace, James Borneman, Jonathan Braun

**Affiliations:** 1 Pathology and Laboratory Medicine UCLA, Los Angeles, CA, USA; 2 The F. Widjaja Family Foundation Inflammatory Bowel and Immunobiology Research Institute, Cedar's Sinai Medical Center, Los Angeles, CA, USA; 3 Biochemistry and Molecular and Cellular Biology, Georgetown University, Washington, DC, USA; 4 Plant Pathology, UC Riverside, Riverside, CA, USA; 5 Biostatistics, UCLA, Los Angeles, CA, USA; 6 Microbiology and Immunology, Stanford University, Palo Alto, CA, USA; 7 Biostatistics, Harvard University, Boston, MA, USA; 8 Molecular and Medical Pharmacology, UCLA, Los Angeles, CA, USA

**Keywords:** Microbiome, Metabolome, Inter-omic analysis

## Abstract

**Background:**

Consistent compositional shifts in the gut microbiota are observed in IBD and other chronic intestinal disorders and may contribute to pathogenesis. The identities of microbial biomolecular mechanisms and metabolic products responsible for disease phenotypes remain to be determined, as do the means by which such microbial functions may be therapeutically modified.

**Results:**

The composition of the microbiota and metabolites in gut microbiome samples in 47 subjects were determined. Samples were obtained by endoscopic mucosal lavage from the cecum and sigmoid colon regions, and each sample was sequenced using the 16S rRNA gene V4 region (Illumina-HiSeq 2000 platform) and assessed by UPLC mass spectroscopy. Spearman correlations were used to identify widespread, statistically significant microbial-metabolite relationships. Metagenomes for identified microbial OTUs were imputed using PICRUSt, and KEGG metabolic pathway modules for imputed genes were assigned using HUMAnN. The resulting metabolic pathway abundances were mostly concordant with metabolite data. Analysis of the metabolome-driven distribution of OTU phylogeny and function revealed clusters of clades that were both metabolically and metagenomically similar.

**Conclusions:**

The results suggest that microbes are syntropic with mucosal metabolome composition and therefore may be the source of and/or dependent upon gut epithelial metabolites. The consistent relationship between inferred metagenomic function and assayed metabolites suggests that metagenomic composition is predictive to a reasonable degree of microbial community metabolite pools. The finding that certain metabolites strongly correlate with microbial community structure raises the possibility of targeting metabolites for monitoring and/or therapeutically manipulating microbial community function in IBD and other chronic diseases.

## Background

The intestinal mucosal surface is the site of a complex orchestration of immunologic, metabolic and ecological forces that drive microbial community structure. In most cases, these forces balance the composition of the gut microbiota with mucosal health, facilitating normal nutrient absorption, local and systemic endocrinology, angiogenesis, epithelial barrier function, brain development, liver function, immune development and gut homeostasis [1-7]. However, the immunological and functional state of the mucosa is influenced by the microbiota, and it is therefore susceptible to detrimental interactions with changes in luminal bacteria [8,9]. The microbial composition is typically well controlled; however, in certain genetically and environmentally susceptible individuals, control of microbial composition is compromised, leading to (or resulting from) clinical manifestations in immune and inflammatory diseases [10-13].

The intestinal mucosal ecosystem harbors an assortment of host factors, microbiota, and metabolites. The microbial ecology in the context of this molecular milieu is an area of intense study, but to this point it has mainly been probed by the potential (versus expressed) functionality represented by the microbial metagenome [14-18]. A central goal and methodologic challenge in human-associated microbial ecology is to identify dietary, metabolic, and host and microbial factors that drive microbial community structure. Recent work by Jansson and colleagues [19,20] and our group [21] indicates that components of the mucosal proteome correlate with certain microbial species and reveals intriguing differences between the potential and expressed biochemical pathways detected in microbial communities *in vivo*[19]. In twin-pair studies, Crohn’s disease-associated differences in fecal metabolites have been detected in parallel with microbial compositional and metagenomic differences in this compartment, and represented biomarkers related to disease state, presumably in part as products of the disease-associated changes in microbial metagenomic function [22-24]. Identification of such relationships is fundamental for interventional strategies to alter microbiota composition in the context of dysbiosis, and have been highlights of recent landmark studies of environment and diet in human fecal microbial composition [11,16,25]. Indeed, direct analysis of metabolic output by and interactions between microbial species is a burgeoning investigative field, but challenging methodologically, particularly *in vivo*[26,27].

**Figure 1:**
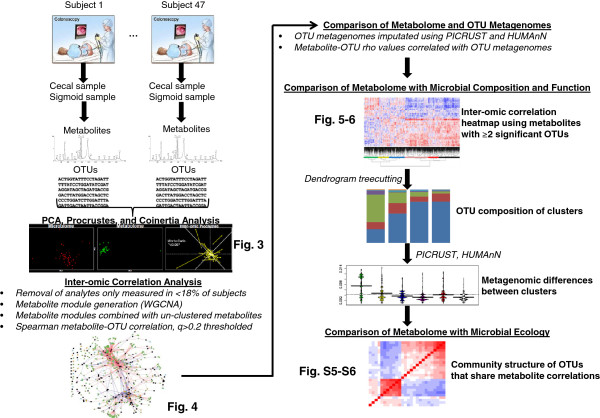
**Procedural schematic.** Endoscopic lavage samples were collected from the cecum and sigmoid colon of each subject. The microbial and metabolic components of each sample were analyzed using Illumina-HiSeq 2000 and ultra performance liquid chromatography (UPLC)-mass spectrometry (MS), respectively. The analytic pipeline thereafter is shown. See methods for additional details. OTU: operational taxonomic unit; PICRUSt, phylotypic investigation of communities by reconstruction of unobserved states; HUMAnN: HMP unified metabolic analysis network; PCA, Principal Component Analysis.

Here, we present our findings with an integrated experimental and bioinformatic approach to identify inter-relationships between microbial composition and metabolism in the human gut (Figure 1). We utilized a top-down strategy to identify metabolic correlates of microbes at the intestinal mucosal surface in a cross-sectional cohort of normal human subjects. Metabolomic and 16S rRNA gene sequencing data were produced from each biospecimen by high-throughput mass spectroscopy and the Illumina Hi-Seq platform, respectively. We further defined the metabolic pathways associated with these microbial communities using metagenomic inference. We characterized the relationship between these pathways and measured metabolites, and we finally catalogued metabolites with potential influence on microbial community structure. Findings primarily analyzed in samples collected from the cecum were validated using paired samples from the sigmoid colon. This study represents one of the first successful integrations of different microbiome components in the adult colonic mucosa.

## Methods

### Sample collection and pre-processing

All enrolled subjects were consented under an approved Institutional Review Board (IRB) protocol from Cedars Sinai Medical Center prior to routine colonoscopy. All subjects underwent bowel preparation with Miralax® prior to colonoscopy. For each sample region, approximately 30ml of sterile water was endoscopically flushed onto the mucosal surface and recollected via aspiration. Samples were obtained from the cecum and sigmoid colon region of each subject. Samples were kept on ice for the duration of the pre-processing that immediately followed sample collection. Samples were centrifuged at 4,000 × g for 10 minutes at 4°C. The supernatant was aliquoted into three 50-ml tubes with equal volumes and frozen at −80°C. The pellets were resuspended in 2 ml of RNAprotect Bacteria Reagent (Qiagen, Valencia, CA, USA), aliquoted into three separate 15-ml conical tubes, centrifuged at 4,000 × g for 10 minutes at 4°C, separated from the supernatant and frozen at −80°C.

### High-throughput 16S analysis

DNA was extracted from 93 samples using the PowerSoil DNA Isolation Kit (MO BIO Laboratories, Carlsbad, CA, USA), and a 30-second beat-beating step using a Mini-Beadbeater-16 (BioSpec Products, Bartlesville, OK, USA). High-throughput sequencing analysis of bacterial rRNA genes was performed using extracted genomic DNA as the templates. One hundred-microliter amplification reactions were performed in an MJ Research PTC-200 thermal cycler (Bio-Rad Inc., Hercules, CA, USA) and contained 50 mM Tris (pH 8.3), 500 μg/ml BSA, 2.5 mM MgCl_2_, 250 μM of each deoxynucleotide triphosphate (dNTP), 400 nM of each primer, 4 μl of DNA template, and 2.5 units JumpStart *Taq* DNA polymerase (Sigma-Aldrich, St. Louis, MO, USA). The PCR primers (F515/R806) targeted a portion of the 16S rRNA gene containing the hypervariable V4 region, with the reverse primers including a 12-bp barcode (Additional file 1) [28]. Thermal cycling parameters were 94°C for 5 minutes; 35 cycles of 94°C for 20 seconds, 50°C for 20 seconds, and 72°C for 30 seconds, and followed by 72°C for 5 minutes. PCR products were purified using a MinElute 96 UF PCR Purification Kit (Qiagen). DNA sequencing was performed using an Illumina HiSeq 2000 (Illumina, Inc., San Diego, CA, USA). Clusters were created using template concentrations of 1.9 pM and PhiX at 65 K/mm^2^, which is recommended by the manufacturer for samples with uneven distributions of A, C, G and T. One hundred base-sequencing reads of the 5’ end of the amplicons and seven base barcode reads were obtained using the sequencing primers listed in Additional file 1. De-multiplexing, quality control, and operational taxonomic unit (OTU) binning were performed using quantitative insights into microbial ecology (QIIME) [29]. The total initial number of sequencing reads was 70,278,364. Low-quality sequences were removed using the following parameters: Q20, minimum number of consecutive high-quality base calls = 100 bp, maximum number of N characters allowed = 0, maximum number of consecutive low-quality base calls allowed before truncating a read = 3. Numbers of sequences removed using the aforementioned quality control parameters were: barcode errors (5,199,568), reads too short after quality truncation (5,545,570), and too many Ns (38,358). Then, 59,494,868 remaining reads were then used to pick OTUs from the GreenGenes reference database, which automatically bins OTUs at 97% identity, so that the resulting data were compatible with phylotypic investigation of communities by reconstruction of unobserved states (PICRUSt) analysis: 1,536,002 reads were discarded during OTU picking due to alignment failure. After OTU picking, 57,958,866 reads remained.

### Metabolomic analysis

#### Solid phase extraction (SPE)

Before cecum and sigmoid lavage aliquots were subjected to metabolomic analysis, they were cleaned with SPE due to the presence of a polymer presumably derived from bowel preparation (bowel preparation often involves polyethylene glycol). The SPE protocol was adopted, modified and made compatible for the downstream mass spectrometry (MS) analysis. MCX cartridges (Waters Corp. Milford, MA, USA) were conditioned with methanol and phosphoric acid prior to use. Each sample was diluted 1:2 in 2% phosphoric acid and loaded on to the MCX cartridge. Samples were incubated with the mix-mod polymer sorbent in the cartridges. The application of vacuum throughout the procedure was kept to the minimum to allow for ample sample/sorbent interaction. The sorbent was then washed with 2% formic acid in water and 10 ml of water. The metabolites were then eluted off the column by subsequent washes with methanol and 5% ammonium hydroxide, dried, and reconstituted in 2% acetonitrile in water.

#### Mass spectrometry analysis

A 5-μl aliquot of extracted metabolites from each sample was injected onto a reverse-phase 50 × 2.1 mm ACQUITY 1.7-μm C18 column (Waters Corp.) using an ACQUITY UPLC system (Waters Corp.) with a gradient mobile phase consisting of 2% acetonitrile in water containing 0.1% formic acid (A) and 2% water in acetonitrile containing 0.1% formic acid (B). Each sample was resolved for 10 minutes at a flow rate of 0.5 ml/minute. The gradient consisted of 100% A for 0.5 minutes, then a ramp of curve 6 to 100% B from 0.5 minutes to 10 minutes. The column eluent was introduced directly into the mass spectrometer by electrospray. MS was performed on a Q-TOF Premier (Waters Corp.) operating in either negative-ion (ESI-) or positive-ion (ESI+) electrospray ionization mode with a capillary voltage of 3,200 V, and a sampling cone voltage of 20 V in negative mode and 35 V in positive mode. The desolvation gas flow was set to 800 L/h and the temperature was set to 350°C. The cone gas flow was 25 L/h, and the source temperature was 120°C. Accurate mass was maintained by introduction of LockSpray interface of sulfadimethoxine (311.0814 (M+H) + or 309.0658 9M-H)−) at a concentration of 250 pg/μl in 50% aqueous acetonitrile and a rate of 150 μl/minute. Data were acquired in centroid mode from 50 to 850 m/z in MS scanning. Centroided and integrated MS data from the UPLC-TOFMS were processed to generate a multivariate data matrix using MarkerLynx (Waters Corp.). The data were normalized to total protein and processed using an array of statistical tools such as R, SIMCA P, and an in-house statistical script. The statistically significant metabolites were putatively identified using several online databses such as HMDB, MMCD, KEGG, and Lipidmaps.

### Bioinformatic analysis

#### Spearman inter-omic correlation analysis

All bioinformatic analysis of cleaned metabolomic and metagenomic data was performed in R. Microbiome and metabolome data from the same samples were merged by subject and thresholded such that analytes measured above background in fewer than 18% subjects were removed from all analyses. The cutoff value of 18% was selected such that similar numbers of observations were eliminated from both the cecum and sigmoid comparisons, and significant inter-omic correlations were not enriched for rare analytes. Inter-omic analysis involved simple Spearman correlation of analyte abundance with calculation of *P*-values using the R function *cor.test*. Spearman correlation was used for inter-omic analysis because it detects more complicated relationships that might otherwise go undetected using other metrics, such as Pearson correlation. Wherever mentioned, the R package *qvalue* was used to generate *q*-values for each spearman correlation. To quantify correlations with individual genera, the following steps were taken. First, OTUs from the correlation data (Additional file 2) were binned by genus assignment. Then, all duplicate metabolic correlations were removed so that even if all OTUs from a genus correlated with a single metabolite it was quantified only as a single interaction.

#### Microbial cluster generation

For heat map-based microbial clustering analysis, heat maps were created of Spearman correlation matrices (without *q*-value thresholding) using the *heatmap.2* function from the *gplots* R package. Hierarchical clustering of bacteria was based on the Euclidian distance metric and the complete method of hierarchical clustering by metabolites (only those with ≥2 significant OTU correlations). Dendrograms were then extracted from the output of that function and cut using the base *cut* function. Cut height was determined by the number of significant modules as defined by prediction strength. We used *k*-means clustering to assess significance in the prediction strength calculations, which has been shown to be a robust strategy for determining optimal cluster number for hierarchical cluster modules [30]. While twenty modules were predicted for the sigmoid data, only six were predicted for the cecal data. To facilitate comparison between regions, we cut the sigmoid dendrogram such that seven (no cut height allowed six) clusters were generated. Prediction strength was performed using the *prediction.strength* function from the *fpc* R package [31]. To determine the similarity of microbial cluster composition, cluster assignment of shared OTUs was examined. Significance of overlapping OTUs between the cecal and sigmoid clusters was determined using one-sided (greater) Fisher’s exact test.

#### Coinertia analysis

Coinertia analysis identifies successive axes of covariance between two datasets involving the same test subjects. Coinertia analysis was performed using the *coinertia* function from the *ade4* R package, applied to eigenvalues of the metabolome and microbiome [32]. The significance of RV scores, which are indicative of global similarity, was estimated using the *RV.rtest* function, which performs a Monte Carlo-based estimation on the sum of eigenvalues from a coinertia analysis.

#### Procrustes analysis

Procrustes analysis analyzes the congruence of two-dimensional shapes produced from superimposition of principal component analyses from two datasets. To remain consistent, we performed Procrustes analysis on the Euclidian distances of eigenvalues for both the microbiome and metabolome using the *Procrustes* function in the *vegan* R package [33].

#### Metabolite module generation

We defined metabolic modules using soft-thresholded Pearson correlation analysis in combination with a topological overlap distance metric and average hierarchical clustering (R package: weighted correlation network analysis (WGCNA)) [34]. Since our goal was to only group metabolites that were highly correlated with each other, we chose to use the more stringent Pearson method for generating modules. Soft-thresholding powers were defined using the *pickSoftThreshold* function of the WGCNA R package. For metabolic module generation, the data were first thresholded such that only metabolites present in at least 18% of samples were included for the module generation pipeline. There are many approaches to such clustering [35-38]. WGCNA uses a measure of shared metabolite neighbors based on topological overlap as input of hierarchical clustering. The height in the dendrogram is a measure of dissimilarity based on the topological overlap matrix; modules are defined as branches of a hierarchical cluster tree [34,39]. WGCNA was attractive for this study since it provides module preservation statistics that allowed us to assess the reproducibility of modules across different data sets; provides a measure of intramodular connectivity that can be used to define intramodular hub genera [40]; and allows us to summarize each module by its module eigenvalue. The resulting modules were then validated using silhouette width and cophenetic distance metrics and compared with two independent module generating approaches: 1) average hierarchical clustering based only on Pearson correlation dissimilarity (1 – Pearson coefficient), and 2) *K* means using unthresholded Pearson correlation analysis. Both independent methods generated strongly similar module composition and distribution.

Using this approach on individual metabolites, the cecum metabolites (soft-thresholding power = 22, minimum module size = 10 metabolites) organized into 20 modules with 121 un-clustered metabolites, while the sigmoid metabolites (soft-thresholding power = 32, minimum module size = 10 metabolites) organized into 15 modules with 170 un-clustered metabolites. For each dataset, the abundance values of the un-clustered analytes were combined with module eigenvalues to facilitate downstream analyses.

#### Putative metabolite identity determination

An in-house script called StandAlone BioIdentifier was used to putatively identify ions based on their biological relevance via incorporation of four major small molecule databases: KEGG, HMDB, LipidMaps, and BioCyc. This metabolomic tool has the unique ability to distinguish mammalian metabolites from those of bacterial and plant origin providing an extra degree of confidence in the ions’ putative IDs. This user-friendly script allows one to choose from several positive and negative adducts at a user-predefined mass tolerance. For our UPLC/MS setup we chose H+ and Na+ adducts for the ESI+ mode and H- and Cl- for the ESI- mode at a predefined mass window of 20 ppm.

#### Quantifying metabolite overlap between the cecum and sigmoid

An R script was written that stringently identified identical metabolites measured in two datasets based on two parameters. The two parameters were defined such that two metabolites must: 1) have a mass difference ≤0.005 m/z, and 2) have a difference in retention time that was ≤0.04 minutes (2.4 seconds).

#### Metagenomic imputation

PICRUSt is a well documented tool designed to impute metagenomic information based on 16S input data (http://picrust.github.com/picrust/). To use the tool to impute metagenomes of microbial OTUs, we created a synthetic OTU table such that each OTU was represented by a single count in a single column. The synthetic table was then input into the PICRUSt pipeline using the terminal interface of a QIIME virtual machine running the Ubuntu operating system. The resulting metagenomic data was input into the HMP unified metabolic analysis network (HUMAnN) pipeline [41] using the same computational platform to sort individual genes into Kyoto Encyclopedia of Genes and Genomes (KEGG) pathways representing varying proportions of each generated metagenome.

#### Defining community structure of metabolite-associated microbes

Abundance data for groups of bacteria that significantly correlated with common metabolites were extracted from the thresholded OTU table (that is, only including bacteria present in at least six samples) and formatted for SparCC, a new tool developed for metagenomic data that simultaneously removes compositional effects while calculating correlation matrices for given OTU tables [42]. For each group of bacteria tested, 1,000 permutations of randomly selected bacteria without replacement (from the same thresholded OTU table) were applied to the same analytic pipeline. For each correlation matrix produced, the average positive and average negative correlation was calculated and compared with the cumulative averages from the permuted datasets. Significance values were the calculated ratios of permuted correlation matrices with stronger positive or negative correlations than the correlation matrix of interest. Technically identical analysis was performed for correlations >0.2 or <−0.2 to supplement the analyses. Total branch lengths were calculated in R using the *compute.brlen* function from the *ape* R package.

## Results

**Figure 2:**
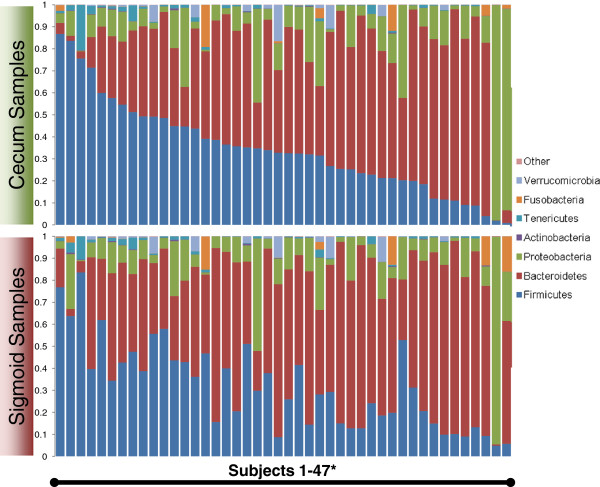
**Phylum-level microbial composition of samples used in this study.** Data are plotted such that cecum and sigmoid samples from the same individual are vertically aligned. ^*^Some samples were excluded to allow vertical alignment by subjects.

To measure the composition, function and interdependence of the colonic microbiome and metabolome, a serial cross-sectional study was performed on human subjects undergoing screening colonic endoscopy: 93 mucosal water-lavage samples from the sigmoid and cecum regions of 47 subjects between the ages of 20 and 83 years (mean 61, SD 14.2) (Table 1). For this pilot study, we included forty-two healthy subjects and five subjects with clinically quiescent Crohn’s disease. We opted to collect mucosal rather than fecal samples, due to the distinct composition and intimate relationship of the mucin-associated microbiota with the colonic epithelium [11,18,43]; lavage sampling permitted efficient recovery of extracellular biosynthetic products present at the mucosal surface [44]. Bacteria were separated from supernatants via centrifugation and the two sample components were analyzed separately for 16S microbiome and metabolome composition [21,44]. However, contaminating polymer, presumably polyethylene glycol from bowel preparation, from metabolic aliquots was removed using SPE (Methods). Cell-free supernatants were analyzed for metabolic content via UPLC-MS with concomitant *in silico* filtering and thresholding (Methods). Cell pellets were analyzed for microbial abundance and composition using the Illumina-HiSeq 2000 platform in combination with the QIIME software suite. Microbial OTUs were rarified to 30,000 reads per sample to reduce noise in downstream analyses. Phylotypic analysis revealed the relative abundance of 2,473 cecum and 2,595 sigmoid GreenGenes reference database-picked OTUs binned at 97% sequence similarity, and thresholded on detection in at least two samples. The represented microbial phylotypic compositions were similar to previously reported colonic mucosal samples and did not indicate significant phylum-level compositional differences between the two colonic regions (Figure 2) [43]. However, biogeographic differences are common in mucosal samples at lower levels of taxonomy [11,45]. Metabolome analysis revealed 649 and 576 metabolite peaks detected in the cecum and sigmoid colon, respectively. Stringent comparison of conserved metabolite masses and retention times between the cecum and sigmoid datasets revealed 342 metabolites present in both the cecum and sigmoid datasets. Furthermore, using putative metabolic IDs from mass, many putative metabolites observed were located at the terminus of metabolic pathways, suggesting enrichment for end products. However, many metabolites had more than one possible putative ID, making precise quantification of end products difficult. However, previous studies have suggested the colonic microbiota contributes to a large representation of metabolic end products [46,47]. Given the large number of metabolites observed, we did not attempt to biochemically validate molecular identities. Instead, we investigated if any inter-omic syntropy could be detected using computational approaches.

**Table 1:** Samples and subjects used for analysis

		**Men**	**Women**	**Total**
**Subjects**	Healthy, number	28	14	42
	Crohn's Disease, number	0	5	5
	Average age, years	62.4	58.7	60.9
**Samples**	Cecum samples, number	27	19	46
	Sigmoid samples, number	28	19	47

### Overview of the measured mucosal microbiome and metabolome

**Figure 3:**
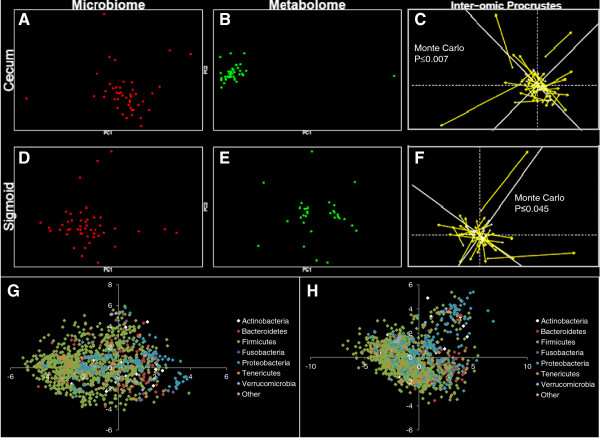
**Principal component, Procrustes and coinertia analysis.** The first column of plots contains microbiome data (red spots) and the second column contains metabolome data (green spots). The first row contains cecal data and the second row contains sigmoid data. Principal component analysis was performed on the cecum microbiome (**A**), cecum metabolome (**B**), sigmoid microbiome (**D**) and sigmoid metabolome (**E**). Inter-omic (**C** and **F**) Procrustes analysis was then performed. Longer lines on Procrustes plots indicate more within-subject dissimilarity of the microbiome and metabolome. Significance values shown were calculated using the *protest* function from the *vegan* R package, which performs repeated symmetric Procrustes analysis to estimate significance. (**G**) and (**H**) show operational taxonomic unit (OTU)-level coinertia analysis. Individual OTUs are plotted based on their cointeria-predicted covariance with the metabolome from the cecum (**A**) and sigmoid (**B**). To reduce noise in this visualization, the data were thresholded such that only OTUs measured above background in ≥18% of samples are shown. Distance from the center is indicative of the strength of covariance.

To determine whether any inter-omic syntropy existed, we first generated two-dimensional principal component distribution plots (PC1 and PC2), with either red (microbiome) or green (metabolome) spots representing each study participant in each data set, and then measured their inter-omic relatedness using Procrustes analysis (Figure 3). Procrustes analysis superimposes and scales principal component plots and allows quantification of non-random congruence between two different measurements from a single group of subjects. To simplify comparisons, Euclidean distances were used in calculating principal components of the microbiome and metabolome constituents. We then performed inter-omic Procrustes analysis on the microbiome and metabolome. Inter-omic Procrustes on cecal samples revealed a strong similarity (Figure 3C: Monte Carlo P ≤0.007), while the sigmoid microbiome and metabolome were less similar, though still significant (Figure 3F: Monte Carlo P ≤0.045). These findings are consistent with recent studies involving the fecal compartment [19,25]. To further confirm this, we also performed coinertia analysis [32]. The coinertia *RV* coefficient is a number between 0 and 1; higher numbers are indicative of more global similarity between two datasets (and for which significance values can be determined). Graphical representation of inter-omic coinertia analysis is shown in Additional file 3. The *RV* scores and Monte Carlo *P*-values were 0.67 and 0.01 for the cecum data, and 0.6 and 0.07 for the sigmoid data, respectively. Excluding subjects <50 years old or subjects with inflammatory bowel disease (IBD) did not increase significance of the inter-omic comparisons using Procrustes or co-inertia analysis, suggesting that age and disease status were not strong drivers of the inter-omic relationship in this cohort (data not shown). Thus, both Procrustes and coinertia metrics indicated that the inter-omic relationship was stronger in the cecum than in the sigmoid. In addition, microbes with the strongest inter-omic covariance, as predicted by the coinertia analysis, are shown in Figure 3G and H. This analysis suggested OTUs from the Firmicutes and Proteobacteria clades were particularly influential to the inter-omic relationship. The corresponding metabolome analysis is available in Additional file 4. Despite the lack of KEGG assignments for metabolites, this analysis indicated metabolites associated with amino acid, porphyrin, and chlorophyll metabolism are important to the inter-omic relationship. Overall, these results supported our hypothesis that significant inter-omic interdependence existed between the metabolome and microbiome. We therefore sought to more clearly define this relationship.

### Construction of metabolic modules based on metabolite co-occurrence

Before proceeding with inter-omic analysis, we first collapsed highly correlated metabolites into modules to streamline and facilitate downstream analyses. Since metabolites associated by biochemical pathway are expected to co-occur, we constructed a network of co-occurrent metabolites, and interrogated the network for modules that might reveal such pathway representation, and would also simplify and strengthen downstream analysis by reducing dimensionality (Methods). The metabolite co-occurrence network was constructed by Pearson correlation, where the edge connecting each pair of nodes was the co-occurrence estimate inferred from the relative abundance profiles of metabolites. Metabolite modules were then identified in the network by an adaptation of WGCNA. The modules generated by WCGNA were validated by independent approaches (Methods). Un-clustered metabolites were combined with module centroids (eigenmetabolites defined as the first singular vector) for each sample. This resulted in a set of 21 and 15 modules and 121 and 170 un-clustered metabolites for the cecum and sigmoid data, respectively. The complete list of metabolites, their module organization, and module dendrograms are available in Additional file 5. In the following phases of this study, datasets containing module eigenvalues and un-clustered metabolites for each separate colonic region were used as the inputs for metabolite-microbial inter-omic analyses.

### Inter-omic network analysis reveals enrichment for shared metabolite associations

**Figure 4:**
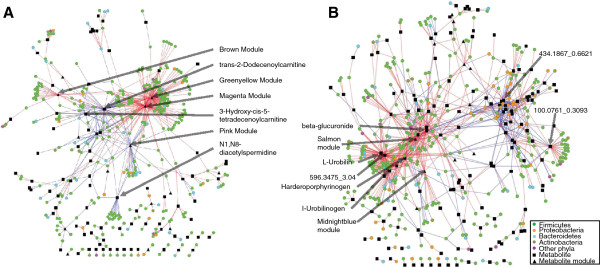
**Inter-omic Spearman correlation networks.** Pair-wise Spearman correlation was calculated for each operational taxonomic unit (OTU) and metabolite (module or un-clustered) pair. Any resulting correlations with *q* ≥0.2 were removed. The resulting correlation networks for the cecum (**A**) and sigmoid (**B**) data are shown. The key provided is applicable to both **A** and **B**. Blue edges represent negative correlation, while red edges represent positive correlation. Selected metabolites and metabolic modules from Table 4 are labeled.

Having found a broad inter-omic relationship between the microbiome and metabolome, we ventured to define the strongest contributors to the relationship. By identifying the analytes with the strongest inter-omic correlations, we could then observe the organization of the resulting interaction network and glean information about the most influential clades and metabolites. Given that approximately 9*10^5^ correlations were calculated for each dataset, we used *q*-values to correct for multiple comparisons, as the Bonferroni *P*-value correction eliminated many strong correlations. Using a threshold of *q* <0.2, we generated a list of 605 and 1,056 unique inter-omic Spearman correlations from the cecum and sigmoid data, respectively. Notable aspects of the resulting networks are shown in Table 2 and graphical representations are shown in Figure 4. On average, metabolite nodes had significantly more edges than OTUs, which was expected given that metabolic pathways are often genomically shared among many organisms. Firmicutes had more *q* <0.2 inter-omic relationships than any other clade, suggesting a more central role in metabolite production (Table 2, row 4). Furthermore, while the ratio of Firmicutes, Bacteroidetes and Tenericutes with significant metabolic correlations was nearly identical between colonic regions, the ratio of Proteobacteria and Actinobacteria with metabolic correlations in the sigmoid was roughly double that of the cecum. This proportional difference remained static when the data were thresholded at *q* <0.1, suggesting that the Proteobacteria and Actinobacteria may play more central metabolic roles in the sigmoid as compared to the cecum.

**Table 2:** Network features of inter-omic analysis

	***q*** **<0.2**		***q*** **<0.1**	
	**Cecum**	**Sigmoid**	**Cecum**	**Sigmoid**
Total interactions, number	605	1056	442	805
Unique microbial nodes, number	338	372	297	342
Unique metabolic nodes, number	95	147	87	142
Ratio of Firmicutes OTUs with metabolic correlations	34%	34%	30%	31%
Ratio of Bacteroidetes OTUs with metabolic correlations	18%	18%	16%	18%
Ratio of Proteobacteria OTUs with metabolic correlations	12%	24%	11%	22%
Ratio of Actinobacteria OTUs with metabolic correlations	25%	54%	20%	54%
Ratio of Tenericutes OTUs with metabolic correlations	24%	24%	19%	24%
Average number of interactions per OTU	1.8	2.8	1.5	2.4
Average number of interactions per metabolite	6.4^**^	7.2^**^	5.1^**^	5.7^**^

^**^Mann–Whitney test, *P* <2 × 10^-16^ for comparison between interactions of operational taxonomic units (OTUs) and metabolites.

To determine the genera with the largest number of significant correlations, OTUs were binned at the genus level and the number of correlated metabolites was quantified for each. Roughly 56% of all OTUs had genus-level resolution, resulting in 255 and 279 unique genera in the cecum and sigmoid data, respectively. However, only eighteen and forty of these had at least two significantly correlated metabolites, suggesting that only a minority of genera had distinguishable metabolic signatures (Table 3). The genera with the largest numbers of correlated metabolites were similar for the cecum and sigmoid. For example, *Clostridium, Blautia, Coprococcus, Bacteroides, Oscillospira, Faecalibacterium, Roseburia* and *Ruminococcus* significantly correlated with the most unique metabolites in both datasets. Therefore, these genera may represent some of the more metabolically unique and/or productive bacteria *in vivo*. Furthermore, several genera had many more interactions in the sigmoid compared with the cecum, including *Mycoplasma, Ralstonia, Aquabacterium, Novosphingobium, Cupriavidus, Actinomyces, Afipia, *and *Collinsella*. Notably, none of these genera were highly abundant in either colonic region but still had ≥10 metabolic interactions in the sigmoid and ≤1 in the cecum, suggesting they might be more metabolically central or active in the sigmoid compared with the cecum. The complete list of *q* <0.2 Spearman correlations is available in Additional file 2.

**Table 3:** **Genera with the most metabolic correlations (*q*** **<0.2) from the cecum and sigmoid data**

**Genus**	**Cecum interactions, number**	**Sigmoid interactions, number**	**Average % abundance in cecum**	**Average % abundance in sigmoid**
*Clostridium*	24	48	5.20	4.61
*Blautia*	24	34	0.58	0.56
*Coprococcus*	13	27	1.05	1.01
*Mycoplasma*	0	23	2.39E-03	1.82E-03
*Ralstonia*	1	22	0.09	0.11
*Bacteroides*	17	21	41.32	43.28
*Oscillospira*	11	19	0.45	0.42
*Aquabacterium*	1	17	0.03	0.04
*Faecalibacterium*	8	16	8.76	7.95
*Roseburia*	8	13	0.95	0.85
*Novosphingobium*	0	13	1.65E-03	2.08E-03
*Cupriavidus*	0	11	2.74E-03	2.08E-03
*Actinomyces*	1	10	7.41E-03	8.01E-03
*Afipia*	0	10	2.65E-04	1.44E-03
*Collinsella*	0	10	0.04	0.05
*Alistipes*	3	9	0.31	0.38
*Stenotrophomonas*	0	9	3.52E-03	2.96E-03
*Parabacteroides*	3	8	3.05	3.88
*Zoogloea*	0	8	1.45E-03	1.39E-03
*Ruminococcus*	11	7	4.20	2.78
*Anaerostipes*	1	7	5.84E-03	7.55E-03
*Oxalobacter*	0	7	2.70E-03	3.60E-03
*Sphingomonas*	0	6	2.48E-03	1.77E-03
*Lachnospira*	2	5	0.35	0.38
*Rothia*	1	5	6.22E-03	5.46E-03
*Streptococcus*	0	5	0.13	0.15
*Desulfovibrio*	0	5	0.06	0.15
*Sutterella*	0	4	2.11	2.11
*Prevotella*	5	3	1.08	1.99
*Mitsuaria*	0	3	9.38E-03	8.71E-03
*Xenophilus*	0	3	2.98E-03	2.91E-03
*Methylotenera*	0	3	3.33E-04	4.68E-04
*Staphylococcus*	0	3	2.05E-03	2.69E-04
*Ramlibacter*	0	3	3.13E-03	3.38E-03
*Bifidobacterium*	3	2	0.06	0.08
*Eubacterium*	2	2	0.16	0.20
*Pantoea*	1	2	1.30E-03	1.38E-03
*Anaerofustis*	0	2	2.20E-04	2.27E-04
*Curvibacter*	0	2	2.20E-04	2.41E-04
*Coprobacillus*	0	2	1.02E-02	1.60E-02
*Dialister*	5	1	0.26	0.27
*Adlercreutzia*	4	1	3.50E-03	6.48E-03
*Actinobacillus*	3	1	0.09	0.04
*Oribacterium*	2	0	7.57E-06	2.04E-03

### Concordance of putative metabolite IDs and functional metagenomic predictions

Having observed strong correlation between individual microbes and metabolites, we were curious about the nature of the correlation. Correlation between microbes and metabolites could arise due to either catabolism/anabolism of metabolites by microbes or stimulation/inhibition of microbial growth by metabolites. To help determine whether catabolic or anabolic reactions might be responsible for any of the observed correlations, we sought to determine whether metabolic associations were concordant with genomic enrichment/depletion of cognate metabolic pathways. The underlying expectation was that observable metabolic differences between organisms would be concomitant with metagenomic enrichments/depletions of the corresponding metabolic pathways.

We were able to impute metagenomes for each OTU using the bioinformatic tool PICRUSt, which allows one to build metagenomes for each OTU using closest-related genomes of cluster OTUs available in the GreenGenes reference database. This bioinformatic method has been productively used in a recent study of functional microbial traits associated with IBD and is robust for large datasets despite the noise introduced through imputation [11]. Accordingly, a metagenome was created for each OTU (representing imputed genes from the closest pre-sequenced GreenGenes OTU) and the relative genomic proportion of each functional pathway was determined using HUMAnN [41].

We then selected all metabolites that were significantly correlated with at least five OTUs (and thus, likely to be more biologically relevant) and had at least one putative molecular ID for this analysis. This resulted in a total of 64 metabolites, and due to multiple possible putative IDs for some metabolites, represented 111 molecules with KEGG pathway associations. For each putative metabolite ID, we generated two vectors: one containing every metabolite-OTU Spearman correlation coefficient for the metabolite in question, and another vector with the imputed metagenomic abundance of the putative KEGGpathway that produces the metabolite in question for every OTU. The two vectors were aligned by OTUs and then compared using Pearson correlation (Additional file 6). The rationale was that bacterial correlation with a single metabolite should be concordant with the corresponding metagenomic abundance of the source metabolic pathway in each bacterium in cases where catabolism or anabolism is the source of the OTU-metabolite correlation. This analysis resulted in 41 significant (Bonferroni *P* <0.05) positive correlations and 31 significant negative correlations; 39 correlations resulted in non-significant *rho* values (Additional file 7). Therefore, given the significance of the relationships, these data suggested some metabolic associations were probably due to microbial catabolic or anabolic reactions. However, more detailed *in vitro* or *in vivo* analyses are necessary to further explore these implications.

### Relationships between the metabolome and microbial composition and function

**Figure 5:**
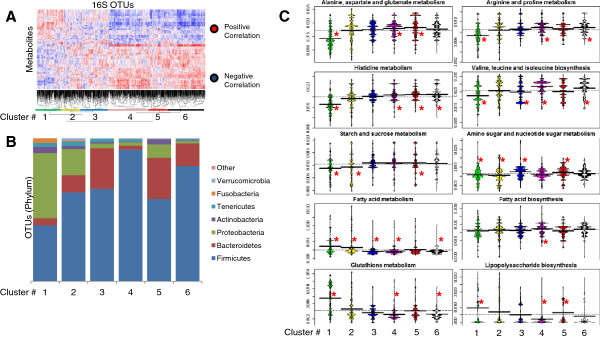
**Cecal microbial clusters.** Heat maps were created that were sorted based on hierarchical clustering of both bacteria and metabolites (**A**). The microbial dendrograms were then cut such that six or seven microbial clusters were generated based on *k*-means prediction strength. The number and horizontal span of each cluster is indicated at the bottom of the dendrogram. The proportion of the phylotypic clades comprising each cluster is depicted for each cluster (**B**). To determine the differences in imputed metagenomic enrichments/depletions of Kyoto Encyclopedia of Genes and Genomes (KEGG) pathways between clusters, the metagenomic composition of each operational taxonomic unit (OTU) was then computed using phylotypic investigation of communities by reconstruction of unobserved states (PICRUSt) and quantitatively analyzed for relative genomic pathway abundance using Unified Metabolic Analysis Network (HUMAnN) and Kruskal-Wallis one-way analysis of variance. Only pathways that significantly varied in at least one cluster (Bonferroni, *P* <0.05) are shown (**C**). Beanplot colors differentiate cluster numbers; ^*^Bonferroni, *P* <0.05.

**Figure 6:**
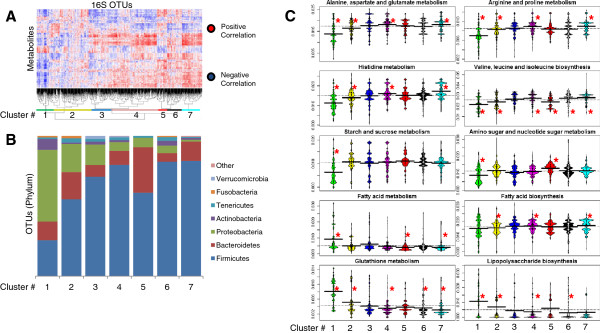
**Sigmoid microbial clusters.** Heat maps were created that were sorted based on hierarchical clustering of both bacteria and metabolites (**A**). The microbial dendrograms were then cut such that six or seven microbial clusters were generated based on *k*-means prediction strength. The number and horizontal span of each cluster is indicated at the bottom of the dendrogram. The proportion of the phylotypic clades comprising each cluster is depicted for each cluster (**B**). To determine the differences in imputed metagenomic enrichments/depletions of Kyoto Encyclopedia of Genes and Genomes (KEGG) pathways between clusters, the metagenomic composition of each operational taxonomic unit (OTU) was then computed using reconstruction of unobserved states (PICRUSt) and quantitatively analyzed for relative genomic pathway abundance using Unified Metabolic Analysis Network (HUMAnN) and Kruskal-wallis one way analysis of variance. Only pathways that significantly varied in at least one cluster (Bonferroni, *P* <0.05) are shown (**C**). Beanplot colors differentiate cluster numbers; ^*^Bonferroni, *P* <0.05.

We then sought to utilize the combined metabolome, imputed metagenome and microbiome data to define relationships between metabolic function and microbial community structure. Our strategy involved stratifying microbes based on their correlations with metabolites that were significantly correlated (*q* <0.2) with two or more OTUs (to reduce noise introduced by stratifying with insignificant metabolites) and analyzing the resulting microbial clusters for phylotypic and functional metabolic enrichments. First, we generated hierarchical cluster-based heat maps of OTUs based on their metabolic Spearman correlations (Figure 5A and 6A). Inspection of these networks revealed clusters of bacteria with similar metabolic associations, reflecting groups of bacteria with dense metabolic similarities. Using prediction strength, we determined that there were ≤6 significant cecal clusters and ≤20 significant sigmoid clusters. However, to allow comparison between the two regions, we cut the hierarchical dendrograms (Additional file 8) such that six cecum clusters and seven sigmoid clusters of bacteria were produced. The phylotypic composition of each resulting cluster was then analyzed (Figure 5B and 6B).

**Figure 7:**
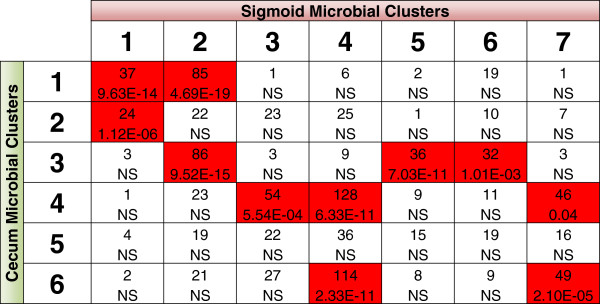
**Compositional comparison of cecum and sigmoid microbial clusters.** The compositional similarity of microbial clusters between the cecum and sigmoid data was assessed by quantifying overlap of cluster assignment for each operational taxonomic unit (OTU) measured from both colonic regions. The top number in each box is the number of shared OTUs in each corresponding cluster and the bottom number is the *P*-value, as determined by Fisher’s exact test. Boxes with significant (Bonferroni, *P* <0.05) overlap are highlighted red. NS, not significant.

Four observations emerged from this analysis at the phylum level. First, microbial clades did not cluster exclusively with themselves; instead, each cluster had a diverse and distinctive representation of microbial phyla. Second, while most clusters contained OTUs from multiple different clades, the Firmicutes, Bacteroidetes and/or Proteobacteria were most prevalent in the composition of each cluster, as expected from their relatively high representation compared to other phyla in the overall microbial community (Figure 2). Furthermore, for both the cecum and sigmoid data the abundance of cluster 1 was dominated by Proteobacteria while the Firmicutes were most abundant in higher-numbered clusters. This most likely reflects the diverse metabolic specialization represented among members of each phylum. Third, this analysis was re-evaluated using finer levels of dendrogram cutting. As expected, this revealed enrichment of dense clusters of phylotypically similar bacteria. However, species-level bacteria did not always cluster together, reflecting potential species-level metabolic divergence (Additional file 9). Such species-level divergence might be expected given the large amount of horizontal gene transfer between intestinal bacteria [48-50]. Fourth, the composition of clusters was highly conserved between the two colonic regions at both the phylum level (Figure 5B and 6B) and OTU level (Figure 7). While cluster composition was not identical between the two regions (for example, cecum cluster 5 was not significantly similar to any sigmoid cluster), groups of bacteria consistently co-segregated with each other, suggesting robust metabolically driven structure that may have broad applicability. Indeed, the differences between the cecum and sigmoid data likely reflect the known biogeographic variation of both metabolites and microbes [11,45,51].

We next attempted to determine the differential metabolic functions represented by each microbial cluster. For this, we chose to focus on imputed metagenomic content of microbes in each cluster due to the rich data made available through PICRUSt. We performed Kruskal-Wallis one-way analysis of variance on all pairs of microbial clusters and imputed metagenomic KEGG pathways and selected some pathways that were significantly different (Bonferroni, *P* <0.05) in at least one cluster. Figures 5C and 6C highlight the genomic representation patterns in the clusters for these pathways. For example, cluster 1 (from both the cecum and sigmoid data) primarily contained Proteobacteria and was enriched for fatty acid metabolism and depleted for several amino acid metabolism pathways. The fact that this microbial cluster contained many Proteobacteria and was enriched for fatty acid metabolism suggests that it may have relevance for IBD, as many studies have shown increases of certain Proteobacteria and decreases of short-chain fatty acids (SCFA) in IBD and other inflammatory diseases [11,52-55]. Conversely, cluster 4 (from both cecum and sigmoid data) were not enriched for fatty acid metabolism but were instead enriched for fatty acid biosynthesis, which would presumably provide SCFA-mediated protection from IBD [56,57]. Indeed, genus level analysis of this cluster revealed enrichment for *Roseburia* and *Faecalibacterium* genera, which have been shown to produce SCFA and are depleted in IBD [11,58-60]. Unsurprisingly, representation of carbon utilization pathways also differentiated the clusters. For example, clusters containing significant amounts of Proteobacteria were depleted for genes encoding starch, sucrose, amino sugar and nucleotide metabolism. Furthermore, genes encoding glutathione metabolism also strongly differentiated the clusters. Among its many functions, glutathione is involved in intracellular oxidative stress control and thus, the differing representation of these genes could be indicative of varying levels of oxidative stress tolerance [61]. In addition, genes involved in lipopolysaccharide (LPS) biosynthesis varied between clusters, likely reflecting the varying composition of Gram-negative bacteria in each cluster.

#### Microbes with shared metabolite associations exhibit significant microbial community structure

One possible source of inter-omic syntropy could be stimulation or inhibition of microbial growth by metabolites. Strong evidence suggests diet can influence microbial composition, but the metabolic processes that select different bacteria remain unclear [16,25,26]. The mechanisms for selection could include any combination of competition, inhibition and niche specialization of bacteria. To try and identify such relationships, we analyzed the microbial abundance data of bacteria that shared at least one correlated metabolite and compared the SparCC [42] correlation structure of the microbial community with that of 1,000 permutations of the same number of randomly selected bacteria. SparCC is a correlation methodology developed specifically for microbial data to eliminate the influence of compositional effects. We reasoned that microbial co-occurrence structure driven by a metabolite (or the metabolic pathway driving production of the metabolite) would manifest as statistically significant increases (cooperation) and decreases (competition) of the average positive and negative SparCC correlation coefficient, respectively. To supplement this analysis, we also examined the number of positive and negative correlation coefficients above 0.2 and below −0.2 for each community (Table 4). We found that approximately 57% of tested metabolite-associated microbial groups had significantly higher positive and lower negative intra-community correlations than randomly selected bacteria.

**Table 4:** Analysis of metabolite-associated microbial communities

**Metabolite mass and retention time**	**Putative ID**	**Colonic region**	**Correlated OTUs, number (*q*** **<0.2)**	**Total branch length**	**Association type**	**Average positive correlation**	**Average negative correlation**	**Number of correlations >0.2**	**Number of correlations <−0.2**
Magenta module	Multiple	Cecum	120	16.58	All positive	0.16^***^	−0.14^***^	2182^***^	1930^***^
Green yellow module	Multiple	Cecum	94	16.48	Mostly positive	0.14^***^	−0.14^***^	1170^***^	1126^***^
342.2631 m/z RT = 4.2272	trans-2-Dodecenoylcarnitine	Cecum	45	11.21	Mostly negative	0.16^***^	−0.14^**^	290^**^	298^***^
Pink module	Multiple	Cecum	31	9.17	Mostly negative	0.13	−0.11	90	58
Brown module	Multiple	Cecum	23	6.81	All positive	0.18^**^	−0.12	88^**^	42
386.2897 m/z RT = 4.3619	3-Hydroxy-cis-5-tetradecenoylcarnitine	Cecum	19	6.18	Mostly negative	0.13	−0.1	28	20
230.1845 m/z RT = 0.4078	N1,N8-diacetylspermidine	Cecum	17	6	All negative	0.32^***^	−0.29^***^	102^***^	84^***^
596.3475 m/z RT = 3.04	?	Sigmoid	61	8.97	All positive	0.14^**^	−0.14^***^	442^**^	406^**^
595.3469 m/z RT = 3.0469	L-Urobilin	Sigmoid	58	10.5	All positive	0.13	−0.12^*^	326	322^*^
593.3309 m/z RT = 3.0247	I-Urobilinogen	Sigmoid	54	9	Mostly positive	0.15^***^	−0.14^***^	384^**^	344^**^
613.3011 m/z RT = 3.0293	Harderoporphyrinogen	Sigmoid	52	9.02	All positive	0.15^***^	−0.14^***^	376^***^	356^***^
605.3348 m/z RT = 3.3185	(23S)-23,25-dihdroxy-24-oxovitamine D3 23-(beta-glucuronide)	Sigmoid	43	10.1	Mostly positive	0.15^*^	−0.13^*^	222^*^	194^*^
591.3203 m/z RT = 3.0304	Mesobilirubinogen	Sigmoid	37	9.03	Mostly positive	0.13	−0.12^*^	152^*^	122
Salmon module	Multiple	Sigmoid	37	8	Mostly positive	0.15^*^	−0.14^**^	184^**^	160^**^
100.0761 m/z RT = 0.3093	2-Hydroxy-2-methylbutanenitrile	Sigmoid	32	8.37	22% Negative, 78% positive	0.21^***^	−0.16^***^	178^**^	196^***^
673.3209 m/z RT = 3.3132	?	Sigmoid	30	7.93	Mostly positive	0.15^*^	−0.14^*^	128^**^	110^**^
434.1867 m/z RT = 0.6621	?	Sigmoid	26	6.54	30% Positive, 70% negative	0.28^***^	−0.21^***^	160^***^	162^***^
619.3474 m/z RT = 3.6896	?	Sigmoid	25	9.13	Mostly positive	0.13	−0.13^*^	76^*^	48
124.0395 m/z RT = 0.3735	Nicotinate	Sigmoid	21	7.47	Mostly negative	0.18^**^	−0.11	58^*^	30
615.3154 m/zRT = 3.0227	Mesobilirubinogen	Sigmoid	18	6.31	All positive	0.1	−0.08	6	2
Midnight blue module	Multiple	Sigmoid	18	6	All positive	0.1	−0.08	14	12

Significance values: ^***^P ≤0.001, ^**^P ≤0.01, ^*^P ≤0.05.

Total branch length is the sum of all branch lengths from Phylogenetic (Newick) trees containing the corresponding operational taxonomic units (OTUs). Retention time (RT) is provided in minutes.

Close examination of the metabolites with multiple *q* <0.2 microbial correlations revealed three classes of metabolites: 1) those with almost exclusively positive correlations with bacteria; 2) those with almost exclusively negative correlations with bacteria; and 3) those with multiple positive and negative correlations with bacteria. Two examples of the latter are shown in Additional file 2, Figure S2 and Additional file 6, Figure S6 and are analyzed further in the Discussion. Unfortunately, due to the partial transitive nature of correlations, we could not conclude that the observed significance of metabolite-associated microbial community structure was ecologically relevant. Lacking tools to disambiguate the transitive features, we can only postulate that metabolites associated with communities with significant community structure may have ecologic influence.

## Discussion

With the advent of next-generation sequencing platforms, a major influx of studies have sought to identify microbial composition differences in various habitats. However, such studies rarely consider environmental variables, such as metabolites or proteins, resulting in incomplete systemic clarity and potentially erroneous assumptions. This study represents one of the first successful attempts to integrate components of the adult gut mucosal ecosystem. We chose to perform analysis on two distinct colonic regions to ensure reproducibility of findings. Notably, all mucosal samples were collected from subjects who had undergone bowel preparation. While standard for both clinical and research endoscopy, bowel preparation is known to alter microbial alpha and beta diversity [62]. Accordingly, such depletion of mucosal microbiota is likely to reduce the scope of detectable inter-omic relationships. However, we reason that the observed relationships are representative of the native mucosa. Also, bowel preparation should result in less dietary and enteric secretion input from the proximal intestine, thereby increasing biogeographic resolution and decreasing noise from dietary metabolites. Nonetheless, it is possible that bowel preparation introduces metabolic changes in the microbial community that elicits non-physiologic inter-omic relationships. Therefore, the scope and quality of these inter-omic relationships merit additional assessment in undisturbed mucosal sites.

This study revealed significant inter-omic structure in both the cecum and sigmoid colon that was independent of age or disease status. While the relationship between the microbiome and metabolome appeared strongest in the cecum by Procrustes and coinertia analysis, a larger number of significant correlations were observed in the sigmoid compared to the cecum, possibly reflecting the known biogeographic differences of both microbes and metabolites [11,45,51]. Despite this biogeographic dissimilarity, we observed significant overlap in findings between the cecum and sigmoid data. While only 342 metabolites were measured in both colonic regions, several observations remained consistent between datasets. As highlighted in Figures 5 to 7, the cecum and sigmoid microbial clusters were very similar in composition and function. Furthermore, as shown in Table 2, the relative ratios of inter-omic correlations were nearly identical at the phylum level. However, some biogeographic differences were observed. For example, the sigmoid data had nearly double the number of significant inter-omic correlations involving Proteobacteria and Actinobacteria, which might suggest differing biogeographic functional roles. Furthermore, using prediction strength, six microbial clusters were predicted for the cecum data while twenty were predicted for the sigmoid, suggesting that microbial function in the sigmoid is much more distinct than in the cecum.

This study also identified microbial clusters that were both metabolically and metagenomically concordant. Using observed metabolic correlations to govern cluster assignment of microbes revealed similarities among diverse groups of bacterial phyla that might not otherwise have phylogenetic or genomic associations. The metabolic relationships defined by these clusters may provide a new avenue to consider *in vivo* microbial function and host response. As noted above, species of Firmicutes, including *Faecalibacterium, Phascolarctobacterium*, and *Roseburia* tend to be depleted in IBD while Proteobacteria species tend to be enriched [11]. All three IBD-depleted genera were substantially confined to cluster 4 of both colonic regions, which was enriched for fatty acid biosynthetic genes and depleted of fatty acid metabolism genes. Fatty acids with the most relevance are SCFA, which are produced as fermentative byproducts. Multiple dietary inputs can be used by bacteria to produce SCFA, including microbial or dietary-derived starch, acetate, lactate, linoleic acid, and fiber [63]. Fatty acid biosynthesis is particularly relevant to the host because SCFA, like butyrate, can 1) act as energy sources for colonocytes; 2) inhibit Nuclear Factor-κB activation in human colonic epithelial cells, resulting in decreased levels of inflammatory cytokines; and 3) stimulate mucin production, which could result in increased barrier protection [64]. Accordingly, reduction in SCFA availability to the host, which could occur if cluster 4 bacteria were depleted, could increase mucosal propensity for and susceptibility to inflammation. However, cluster 4 is also highly enriched for various amino acid metabolic and biosynthetic pathways that could also influence the environmental availability of such molecules and thereby contribute to mucosal homeostasis in ways that have not yet been examined (Figures 5C and 6C). Furthermore, cluster 4 was depleted of genes from the KEGG glutathione metabolism pathway, which includes both biosynthetic and metabolic genes. Glutathione is important for mitigating oxidative stress and acts as a powerful redox buffer [61]. Therefore, depletion of genes involved in production and metabolism of glutathione could indicate that bacteria in cluster 4 were more susceptible to oxidative stress and would therefore be at a selective disadvantage in oxidative inflammatory conditions like those found in IBD. Conversely, cluster 1, which contained many Proteobacteria, was significantly enriched with genes from the glutathione metabolism pathway, which could explain why such bacteria tend to more abundant in IBD [65]. Therefore, grouping metabolically similar bacteria into clusters aids functional analyses by 1) reducing the dimensionality of data; 2) allowing assignment of potential functions to bacteria that might not be culturable *in vitro*; and 3) defining collective relationships between bacteria that might not be overtly related by phylogenetic sequences.

Another central finding of this study was the rich network of significant correlations between the microbiome and metabolome. Such correlation structure likely arises from a combination of two general processes: 1) catabolism and anabolism of metabolites by microbes, and 2) stimulation and inhibition of microbial growth by metabolites. Indeed, it is widely accepted that dietary alteration is accompanied by shifts in gut microbiome composition and that microbial composition influences the intestinal metabolome [16,25,26]. However, the metabolites and metabolic pathways involved in such processes are unknown. Therefore, while it is difficult to conclusively assign cause and effect to correlation data, a central goal of this study was to determine whether bioinformatic signatures of either process could be detected.

To observe whether catabolic or anabolic reactions contributed to the inter-omic correlation structure, KEGG pathway representation of imputed OTU metagenomes was correlated with the correlation coefficients from the pair-wise microbe-metabolite comparisons. While roughly half of these comparisons had significant positive correlation coefficients, indicating concordance between observed metabolite abundance and metagenomic abundance, a large proportion of correlations were insignificant or significantly negative (Additional file 6). While the mechanisms remain unclear, multiple possibilities exist for the differing directionality of observed correlations. For example, a metabolic end product might have a positive correlation with the OTU (and thus the originating pathway) that produces and exports it, but a negative correlation with the OTU (and thus the originating pathway) that imports and processes it in a downstream pathway. Unfortunately, this also means that some metabolites might have less significant correlation curves due to organisms that encode enzymes producing such metabolites, but are not correlated with the metabolites because they are not exported, which was required for us to measure the association. Given the immature understanding of gut metabolic pathways and the imperfect nature of putative metabolite ID picking, we were not able to resolve this issue. Regardless, these data suggest that our predicted metagenomic and putative metabolite ID data were concordant. This was an important finding because defining metabolite IDs using biochemical methods is subject to numerous limitations that could be simplified with metagenomic data. To try and detect signatures of microbial inhibition or stimulation, we attempted to quantify the significance of community structure between microbes with shared metabolite correlations. While significant community structure was observed between microbes, we were unable to deconvolute the transitive effects of correlation and thus, could not conclude that metabolite-mediated microbial stimulation/inhibition occurred. However, we provided two examples of communities that appeared to have exceptional structure, even compared against other metabolite-associated microbial communities. Additional file 10 shows the structure of a microbial community that was defined by common correlation with a cecal metabolite (mass = 230.1845, retention time = 0.4078 minutes). This metabolite negatively correlated with 17 bacteria. When correlated with each other, these bacteria formed two tight clusters with extremely well-defined co-exclusion structure. An appealing explanation for this type of behavior is that the two communities actively compete with each other for consumption of the metabolite. The metabolite-associated community in Additional file 11 involved a sigmoid metabolite (mass = 434.1867, retention time = 0.6621 minutes). This metabolite was negatively correlated with one Tenericute and eighteen Proteobacteria OTUs and positively correlated with seven Firmicutes OTUs. When correlated with each other, these bacteria also formed two distinct communities that were extremely co-exclusive; one community contained all the Firmicutes, and the other community contained most of the Proteobacteria. This behavior could be indicative of a Firmicutes-produced metabolite that is inhibitory to Proteobacteria. While it is possible that the observed structure was due to the transitive nature of the correlations, these observations would seem to suggest that metabolites might be driving microbial community structure and may directly or indirectly modulate inter-species competition.

Regardless of the mechanism of correlation, the strong correlation between individual metabolites and microbes has numerous potential implications for future innovations. For example, strong correlative relationships may have value as biomarkers for individual or groups of analytes. An obvious application would be the use of specific metabolites as indicators of the presence of certain bacteria, which would presumably be faster than culture- or sequence-based approaches. Furthermore, knowledge of the relationships between metabolites and bacteria may prove useful in either direct (therapeutic) or indirect (dietary) interventions for chronic disorders with microbial compositional shifts, such as IBD.

## Conclusions

The data presented here reveal significant interdependence of the mucosal metabolome and microbiome. Evidence was presented that suggests the microbiome and metabolome have bi-directional influence, with bacteria influencing metabolite composition and metabolites contributing to microbial community architecture. The results also suggest that metabolites should be more deeply interrogated as direct mediators of microbial-associated disease activity and that metabolites may be a direct target for monitoring and therapeutically manipulating microbial community function in IBD and other microbiome-associated intestinal diseases.

## Abbreviations

Bp: Base pair; BSA: Bovine serum albumin; CD: Crohn’s disease; dNTP: Deoxynucleotide triphosphate; HUMAnN: HMP unified metabolic analysis network; IBD: Inflammatory bowel disease; KEGG: Kyoto Encyclopedia of Genes and Genomes; LPS: Lipopolysaccharide; MS: Mass spectrometry; OTU: Operational taxonomic unit; PCR: Polymerase chain reaction; PICRUSt: Phylotypic investigation of communities by reconstruction of unobserved states; UC: Ulcerative colitis; UPLC: Ultra performance liquid chromatography; SCFA: Short-chain fatty acid; SPE: Solid phase extraction; QIIME: Quantitative Insights into Microbial Ecology; WGCNA: Weighted correlation network analysis.

## Competing interests

The authors declare that they have no competing interests.

## Authors’ contributions

IM and MG contributed equally to this work. AF, JBo, DM, TG, JS and JBr designed the study format. DM coordinated the sample collection. IM and MT pre-processed samples and coordinated sample storage and distribution. IM, JBr, CH and MT designed the analytic strategies. JBr led biweekly conference calls with AF, MG, JBo, TG, JS, DM, MT, and IM to coordinate analysis. PR, JW and JBo generated sequencing data. MG and AF generated metabolomic data. IM, MG, MT, ES, JBo, PR, SH, CH and JBr analyzed data. IM, MT and MG performed computational analysis. All authors interpreted data. IM, MG, AF, JBo and JBr drafted the manuscript. All authors read and approved the final manuscript.

## Supplementary Material

Additional file 1 **Primers used for the V4 rRNA PCR and sequencing analysis.** Contains three supplemental tables containing: (1) Reverse PCR primers used in the Illumina-based high-throughput sequence analysis of bacterial 16S rRNA genes; (2) Forward PCR primer used in the Illumina-based high-throughput sequence analysis of bacterial 16S rRNA genes; and (3) Sequencing primers used in the Illumina-based high-throughput sequence analysis of bacterial 16S rRNA genes. PCR, polymerase chain reaction. Click here for file

Additional file 2 **Inter-omic metabolome versus microbiome correlation analysis for cecum and sigmoid data.** A list of *q* <0.2 significant correlations between the microbiome and metabolome is included with Spearman *Rho* values, *P*-values and *q*-values for each comparison. Data from the cecum and sigmoid data are included in separate worksheets. Click here for file

Additional file 3 **Coinertia analysis.** Coinertia analysis was performed to compare the global similarity of the microbiome and metabolome. The cecum (**A**) and sigmoid (**B**) coinertia analysis of the microbiome and metabolome measured from the same samples are shown. As with Procrustes analysis, longer lines indicate greater dissimilarity. Click here for file

Additional file 4 **Metabolite-level coinertia analysis.** Individual metabolites are plotted based on their coinertia-predicted covariance with the microbiome from the cecum (**A**) and sigmoid (**B**). To reduce noise, data were first thresholded such that only metabolites measured above background in ≥18% of samples were analyzed. Distance from the center is indicative of the strength of covariance. Technical limitations limited assignment of putative IDs, and thus Kyoto Encyclopedia of Genes and Genomes (KEGG) pathways, to metabolites, so only a minority of metabolites are labeled.) Click here for file

Additional file 5 **Metabolites and module colors from cecum and sigmoid.** Module assignment for each metabolite is provided. Since the IDs of the measured metabolites are only putative, the technical information is provided for each metabolite, including the ion mode used to detect the metabolite, the measured m/z mass, and retention time (in minutes). Metabolites that were not grouped into modules are designated as un-clustered. Module dendrograms are included after each list of cecum and sigmoid metabolites. Click here for file

Additional file 6 **Correlations of inter-omic data and metagenomic data.** Correlations values for each microbial-metabolite pair were compared with metagenomic data for each respective operational taxonomic unit (OTU), such that metabolite-associated pathways could be tested against metagenomic data. The rationale was that bacterial correlations with a single metabolite should be concordant with the corresponding metagenomic abundance of the source metabolic pathway in each bacterium in cases where catabolism or anabolism is the source of the OTU-metabolite correlation. Each of the 111 tested correlations is shown from highest to lowest Pearson *rho* value. Correlations with significant Bonferroni-corrected *P*-values (*P* <0.05) are shaded in red. Insignificant correlations are shaded in grey. Click here for file

Additional file 7 **Comparison of metagenomic abundance data with correlated metabolite data.** A list of metabolites is included with the colonic region detected, the module name (if the metabolite was clustered into a module), putative KEGG identifiers for each metabolite and the KEGG pathway names from which the metabolites are produced. For each putative ID, we generated two vectors: one containing every metabolite-operational taxonomic unit (OTU) Spearman correlation coefficient for the metabolite in question, and another vector with the imputed metagenomic abundance of the putative Kyoto Encyclopedia of Genes and Genomes (KEGG) pathway that produces the metabolite in question for every OTU. The two vectors were then aligned by OTUs and then compared using Pearson correlation. The Pearson correlation value is represented in the column entitled Metabolite-metagenome correlation. Uncorrected and corrected (Bonferroni) *P*-values are also provided. Click here for file

Additional file 8 **generation of microbial clusters from metabolically driven hierarchical clusters.** Dendrograms of the cecum (**A**) and sigmoid (**B**) are shown with red lines indicating the height at which each dendrogram was cut. Cut heights were selected based on prediction strength to yield six (cecum) or seven (sigmoid) clusters. Click here for file

Additional file 9 **Microbial cluster designation for each operational taxonomic unit (OTU).** Two worksheets are provided containing OTUs and the clusters for which they were assigned from the cecum (first worksheet) and sigmoid (second worksheet) data. Taxonomic information is also provided for each OTU along with GreenGenes ID numbers. Click here for file

Additional file 10 **Microbial community structure potentially driven by microbial competition for a metabolite.** A heat map generated from a SparCC-correlated matrix of operational taxonomic units **(**OTUs) that were all found to significantly negatively correlate with a single metabolite (mass = 230.1845, retention time = 0.4078 minutes). The heat map depicts two strongly co-occurrent clusters of bacteria that display co-exclusive behavior. The microbial IDs are shown to the left of each corresponding row in the heat map. This behavior could be explained by competition by the two communities for the associated metabolite. Click here for file

Additional file 11 **Microbial community structure potentially driven by an inhibitory metabolite.** A heat map generated from a SparCC-correlated matrix of operational taxonomic units (OTUs) is shown; they were all either positively or negatively correlated with a single metabolite (mass = 434.1867, retention time = 0.6621 minutes). OTUs from the Firmicutes clade were exclusively positively associated with the metabolite, while OTUs from the Proteobacteria clade were exclusively negatively associated with the metabolite. The SparCC heat map shows these two phylogenetically distinct communities were extremely co-exclusive. The microbial IDs are shown to the left of each corresponding row in the heat map. One potential explanation for this phenomenon is if the metabolite is an inhibitory metabolite that specifically targets members of the Proteobacteria clade that is produced by OTUs from the Firmicutes clade. Click here for file
