# Supplementary figures and images for: Integrative analysis of the microbiome and metabolome of the human intestinal mucosal surface reveals exquisite inter-relationships
Source: Microbiome. 2013 Jun 5;1:17. doi: 10.1186/2049-2618-1-17 (PMC3971612; doi:10.1186/2049-2618-1-17)

**A**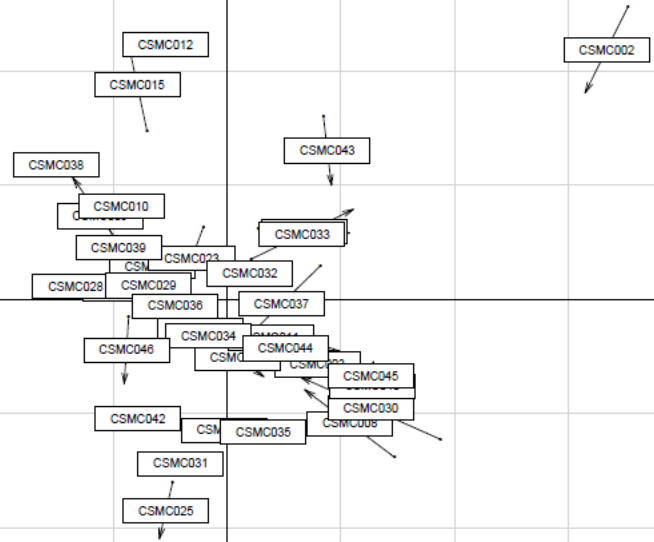**B**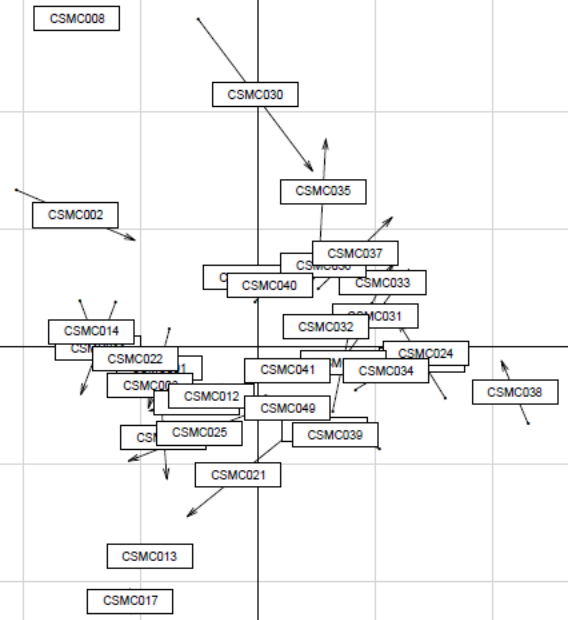

Supplement: Additional file 3 — Coinertia analysis. Coinertia analysis was performed to compare the global similarity of the microbiome and metabolome. The cecum (A) and sigmoid (B) coinertia analysis of the microbiome and metabolome measured from the same samples are shown. As with Procrustes analysis, longer lines indicate greater dissimilarity. [file 2049-2618-1-17-S3.pdf]

**A**

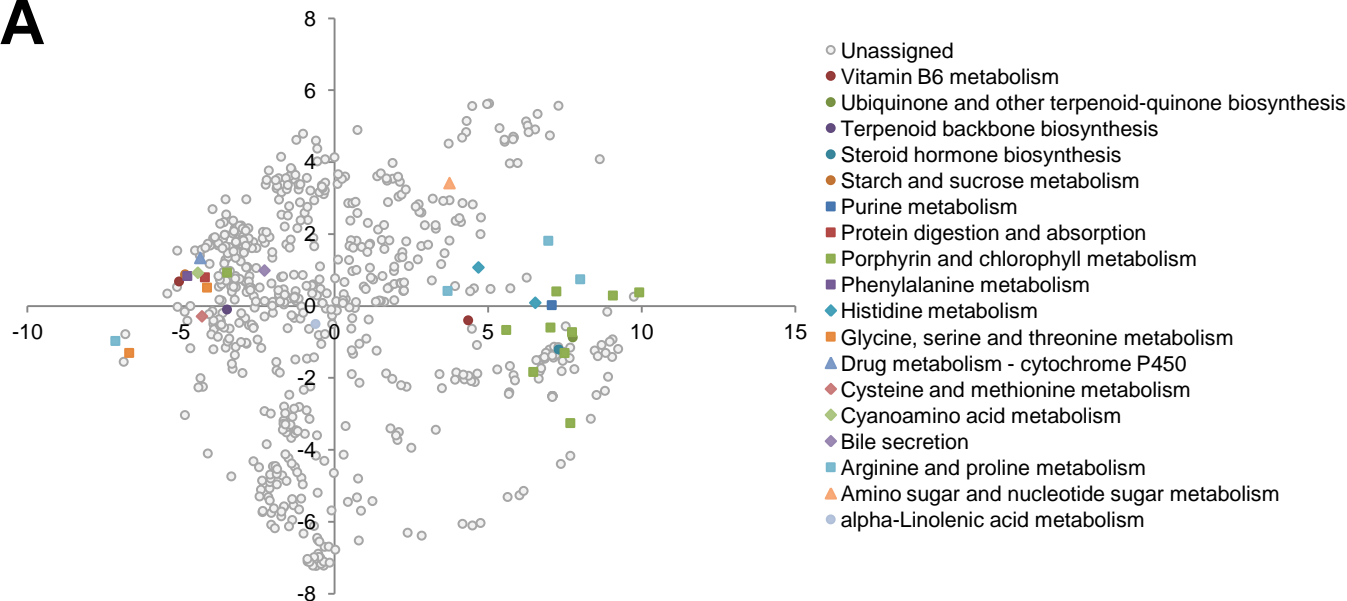

**B**

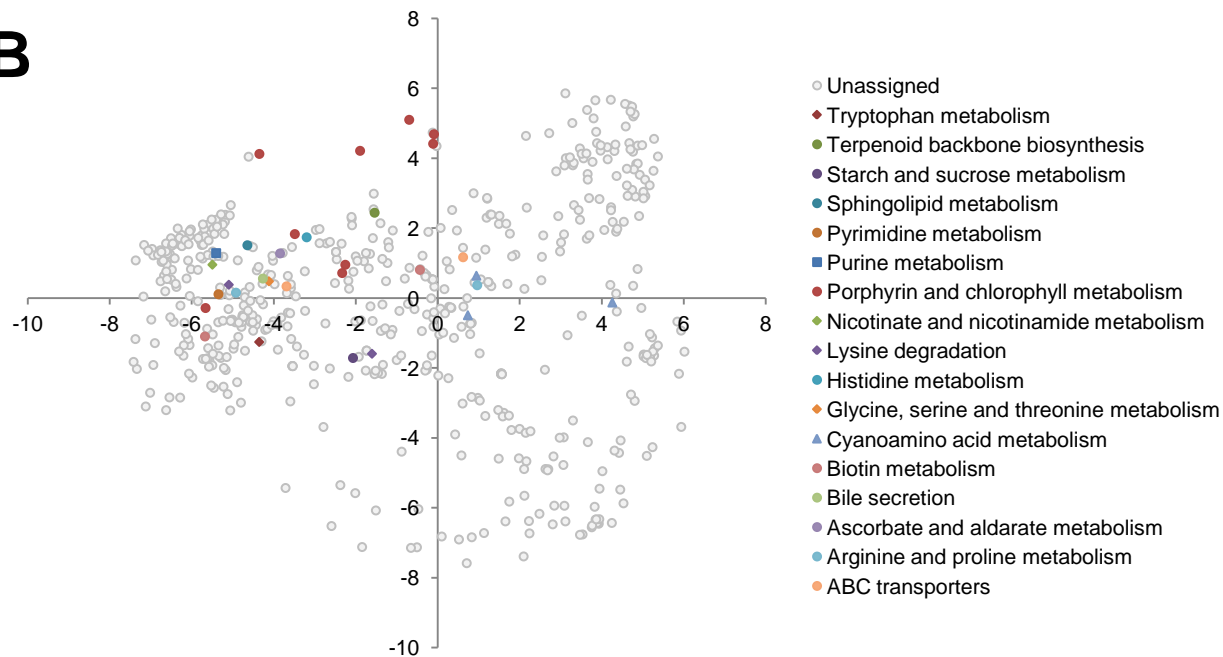

Supplement: Additional file 4 — Metabolite-level coinertia analysis. Individual metabolites are plotted based on their coinertia-predicted covariance with the microbiome from the cecum (A) and sigmoid (B). To reduce noise, data were first thresholded such that only metabolites measured above background in ≥18% of samples were analyzed. Distance from the center is indicative of the strength of covariance. Technical limitations limited assignment of putative IDs, and thus Kyoto Encyclopedia of Genes and Genomes (KEGG) pathways, to metabolites, so only a minority of metabolites are labeled.) [file 2049-2618-1-17-S4.pdf]

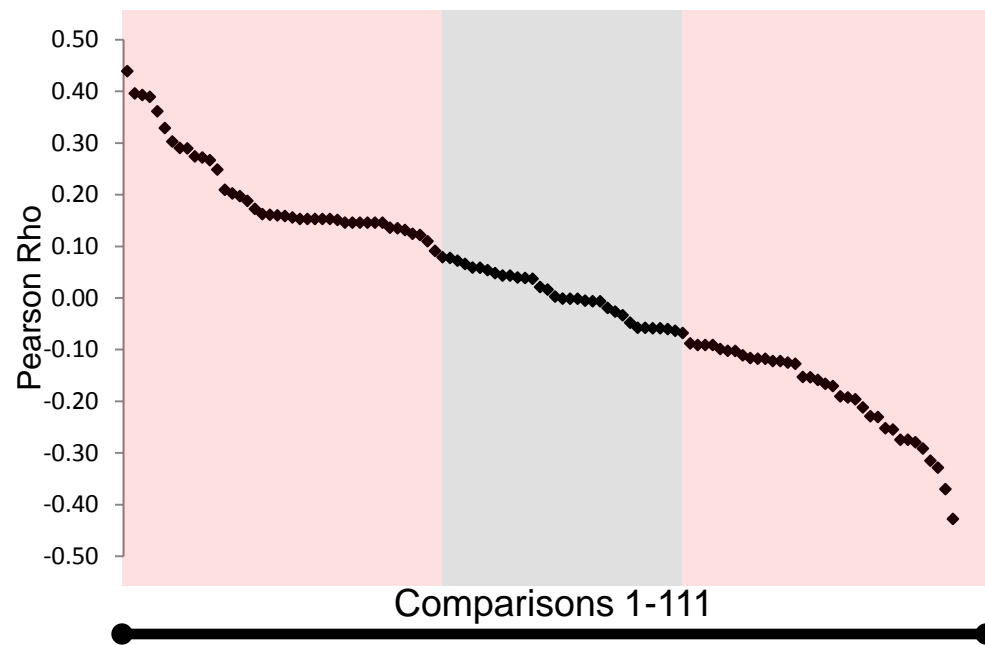

Supplement: Additional file 6 — Correlations of inter-omic data and metagenomic data. Correlations values for each microbial-metabolite pair were compared with metagenomic data for each respective operational taxonomic unit (OTU), such that metabolite-associated pathways could be tested against metagenomic data. The rationale was that bacterial correlations with a single metabolite should be concordant with the corresponding metagenomic abundance of the source metabolic pathway in each bacterium in cases where catabolism or anabolism is the source of the OTU-metabolite correlation. Each of the 111 tested correlations is shown from highest to lowest Pearson rho value. Correlations with significant Bonferroni-corrected P-values (P <0.05) are shaded in red. Insignificant correlations are shaded in grey. [file 2049-2618-1-17-S6.pdf]

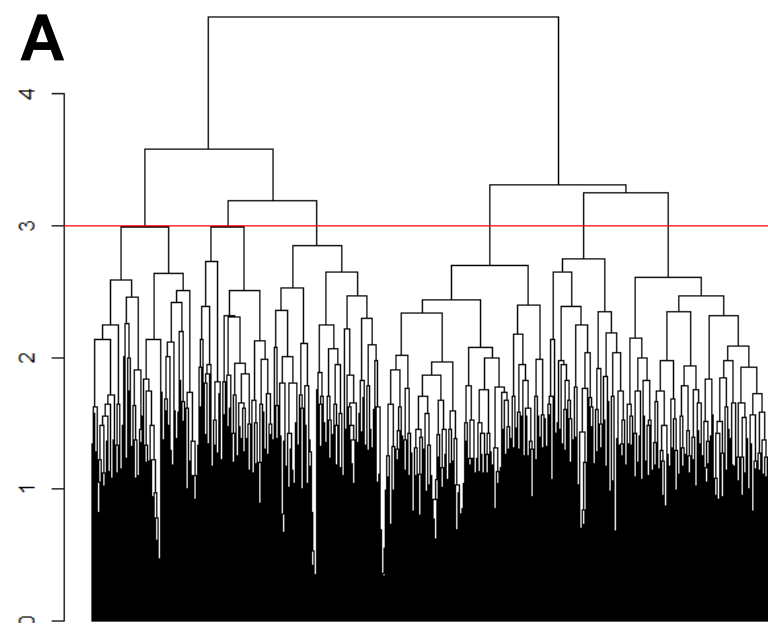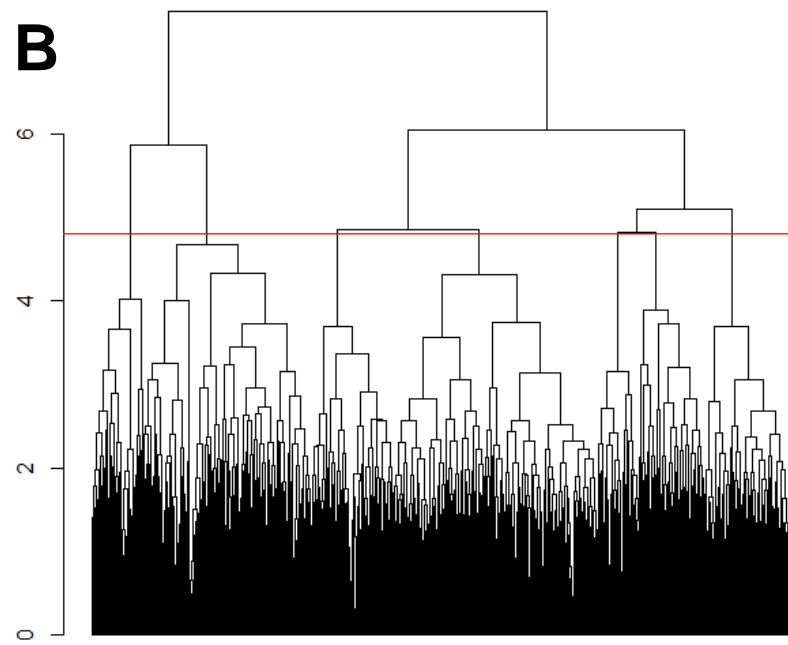

Supplement: Additional file 8 — generation of microbial clusters from metabolically driven hierarchical clusters. Dendrograms of the cecum (A) and sigmoid (B) are shown with red lines indicating the height at which each dendrogram was cut. Cut heights were selected based on prediction strength to yield six (cecum) or seven (sigmoid) clusters. [file 2049-2618-1-17-S8.pdf]
