# Supplementary material for: Integrative analysis of the microbiome and metabolome of the human intestinal mucosal surface reveals exquisite inter-relationships
Source: Microbiome. 2013 Jun 5;1:17. doi: 10.1186/2049-2618-1-17 (PMC3971612; doi:10.1186/2049-2618-1-17)
Supplement: Additional file 5 — Metabolites and module colors from cecum and sigmoid. Module assignment for each metabolite is provided. Since the IDs of the measured metabolites are only putative, the technical information is provided for each metabolite, including the ion mode used to detect the metabolite, the measured m/z mass, and retention time (in minutes). Metabolites that were not grouped into modules are designated as un-clustered. Module dendrograms are included after each list of cecum and sigmoid metabolites. [file 2049-2618-1-17-S5.pdf]

Cecum Metabolites				
Metabolite	Dection Ion Mode	Mass (m/z)	Retention Time (Minutes)	Module
M.452.2766_5.7848	Negative	452.2766	5.7848	black
M.229.0979_1.7973	Negative	229.0979	1.7973	black
M.496.2678_5.8887	Negative	496.2678	5.8887	black
M.520.2661_5.7818	Negative	520.2661	5.7818	black
M637.306_7.1612	Positive	637.306	7.1612	black
M282.2785_5.104	Positive	282.2785	5.104	black
M454.2914_5.7787	Positive	454.2914	5.7787	black
M654.3315_7.1609	Positive	654.3315	7.1609	black
M617.3339_4.4435	Positive	617.3339	4.4435	black
M659.2866_7.1597	Positive	659.2866	7.1597	black
M669.3322_7.1593	Positive	669.3322	7.1593	black
M682.3624_7.1589	Positive	682.3624	7.1589	black
M300.2886_5.3907	Positive	300.2886	5.3907	black
M302.3055_5.2441	Positive	302.3055	5.2441	black
M691.3142_7.1588	Positive	691.3142	7.1588	black
M436.2822_5.777	Positive	436.2822	5.777	black
M669.3316_7.4436	Positive	669.3316	7.4436	black
M498.2812_5.8912	Positive	498.2812	5.8912	black
M686.359_7.1579	Positive	686.359	7.1579	black
M142.0266_7.0748	Positive	142.0266	7.0748	black
M711.3371_6.6123	Positive	711.3371	6.6123	black
M.173.0814_2.3546	Negative	173.0814	2.3546	blue
M.112.985_0.2697	Negative	112.985	0.2697	blue
M.194.0814_4.7286	Negative	194.0814	4.7286	blue
M.248.9596_0.2681	Negative	248.9596	0.2681	blue
M.242.1754_3.5532	Negative	242.1754	3.5532	blue
M.110.9752_0.3297	Negative	110.9752	0.3297	blue
M.213.0146_3.1106	Negative	213.0146	3.1106	blue
M.316.9475_0.268	Negative	316.9475	0.268	blue
M.180.972_0.2662	Negative	180.972	0.2662	blue
M.174.9545_0.2536	Negative	174.9545	0.2536	blue
M.154.9731_0.2579	Negative	154.9731	0.2579	blue
M.384.9342_0.2685	Negative	384.9342	0.2685	blue
M.452.9221_0.2698	Negative	452.9221	0.2698	blue
M.369.151_2.3549	Negative	369.151	2.3549	blue
M.520.9089_0.2698	Negative	520.9089	0.2698	blue
M.588.8976_0.2698	Negative	588.8976	0.2698	blue
M118.1219_0.3166	Positive	118.1219	0.3166	blue
M263.1379_1.6619	Positive	263.1379	1.6619	blue
M249.1564_1.5684	Positive	249.1564	1.5684	blue
M126.0907_0.3895	Positive	126.0907	0.3895	blue
M97.0753_0.4419	Positive	97.0753	0.4419	blue
M86.0594_0.5169	Positive	86.0594	0.5169	blue
M130.1592_1.6732	Positive	130.1592	1.6732	blue
M118.0861_0.3118	Positive	118.0861	0.3118	blue

M295.1646_2.1273	Positive	295.1646	2.1273	blue
M227.1742_1.5686	Positive	227.1742	1.5686	blue
M261.1305_1.4714	Positive	261.1305	1.4714	blue
M84.9594_0.2467	Positive	84.9594	0.2467	blue
M191.0761_0.266	Positive	191.0761	0.266	blue
M217.1034_1.202	Positive	217.1034	1.202	blue
M229.0676_1.7599	Positive	229.0676	1.7599	blue
M349.1816_1.7925	Positive	349.1816	1.7925	blue
M270.1484_2.9726	Positive	270.1484	2.9726	blue
M305.1562_1.644	Positive	305.1562	1.644	blue
M393.2082_1.9204	Positive	393.2082	1.9204	blue
M141.1128_0.276	Positive	141.1128	0.276	blue
M245.1354_2.0162	Positive	245.1354	2.0162	blue
M132.0022_0.245	Positive	132.0022	0.245	blue
M192.1582_0.3595	Positive	192.1582	0.3595	blue
M371.2258_1.9204	Positive	371.2258	1.9204	blue
M437.2338_2.0324	Positive	437.2338	2.0324	blue
M327.2001_1.7911	Positive	327.2001	1.7911	blue
M481.2602_2.1338	Positive	481.2602	2.1338	blue
M90.9762_0.2699	Positive	90.9762	0.2699	blue
M476.3049_2.1334	Positive	476.3049	2.1334	blue
M104.0085_0.2561	Positive	104.0085	0.2561	blue
M415.2521_2.03	Positive	415.2521	2.03	blue
M200.0399_0.2697	Positive	200.0399	0.2697	blue
M432.2778_2.0279	Positive	432.2778	2.0279	blue
M229.0672_1.5077	Positive	229.0672	1.5077	blue
M459.2793_2.1311	Positive	459.2793	2.1311	blue
M579.8382_2.9719	Positive	579.8382	2.9719	brown
M557.8247_2.9379	Positive	557.8247	2.9379	brown
M577.3593_2.9701	Positive	577.3593	2.9701	brown
M520.3313_2.2219	Positive	520.3313	2.2219	brown
M564.3575_2.3058	Positive	564.3575	2.3058	brown
M535.8107_2.8918	Positive	535.8107	2.8918	brown
M599.3749_3.0052	Positive	599.3749	3.0052	brown
M525.2869_2.222	Positive	525.2869	2.222	brown
M555.3469_2.9377	Positive	555.3469	2.9377	brown
M601.8516_3.0048	Positive	601.8516	3.0048	brown
M608.3827_2.3815	Positive	608.3827	2.3815	brown
M569.3127_2.3045	Positive	569.3127	2.3045	brown
M621.3879_3.0369	Positive	621.3879	3.0369	brown
M513.7975_2.859	Positive	513.7975	2.859	brown
M533.3333_2.8911	Positive	533.3333	2.8911	brown
M152.0559_0.3227	Positive	152.0559	0.3227	brown
M643.4018_3.0595	Positive	643.4018	3.0595	brown
M491.7843_2.8141	Positive	491.7843	2.8141	brown
M652.409_2.4505	Positive	652.409	2.4505	brown
M613.3395_2.3791	Positive	613.3395	2.3791	brown

M511.3204_2.8616	Positive	511.3204	2.8616	brown
M480.7938_2.8135	Positive	480.7938	2.8135	brown
M469.7712_2.7739	Positive	469.7712	2.7739	brown
M502.8066_2.8569	Positive	502.8066	2.8569	brown
M458.7811_2.7697	Positive	458.7811	2.7697	brown
M524.8204_2.891	Positive	524.8204	2.891	brown
M687.4247_3.1245	Positive	687.4247	3.1245	brown
M489.3078_2.8133	Positive	489.3078	2.8133	brown
M546.8339_2.9417	Positive	546.8339	2.9417	brown
M696.435_2.5036	Positive	696.435	2.5036	brown
M439.242_2.7248	Positive	439.242	2.7248	brown
M568.8465_2.971	Positive	568.8465	2.971	brown
M665.4115_3.0931	Positive	665.4115	3.0931	brown
M657.3674_2.4504	Positive	657.3674	2.4504	brown
M160.1323_0.3297	Positive	160.1323	0.3297	cyan
M124.0389_0.3603	Positive	124.0389	0.3603	cyan
M553.9932_3.261	Positive	553.9932	3.261	cyan
M524.6426_3.224	Positive	524.6426	3.224	cyan
M539.3173_3.2442	Positive	539.3173	3.2442	cyan
M703.5753_8.0051	Positive	703.5753	8.0051	cyan
M509.9672_3.2031	Positive	509.9672	3.2031	cyan
M504.292_3.2037	Positive	504.292	3.2037	cyan
M518.9659_3.224	Positive	518.9659	3.224	cyan
M537.6684_3.2445	Positive	537.6684	3.2445	cyan
M533.644_3.2525	Positive	533.644	3.2525	cyan
M531.9926_3.2525	Positive	531.9926	3.2525	cyan
M709.4391_3.1516	Positive	709.4391	3.1516	cyan
M393.2985_8.154	Positive	393.2985	8.154	green
M550.6314_8.125	Positive	550.6314	8.125	green
M413.2665_8.1497	Positive	413.2665	8.1497	green
M459.3019_8.253	Positive	459.3019	8.253	green
M337.1043_4.9611	Positive	337.1043	4.9611	green
M149.0231_8.1481	Positive	149.0231	8.1481	green
M504.36_8.2537	Positive	504.36	8.2537	green
M783.5752_8.1475	Positive	783.5752	8.1475	green
M371.3153_8.1475	Positive	371.3153	8.1475	green
M129.0553_8.1484	Positive	129.0553	8.1484	green
M149.0234_5.9534	Positive	149.0234	5.9534	green
M301.1411_5.9536	Positive	301.1411	5.9536	green
M610.1836_8.8022	Positive	610.1836	8.8022	green
M763.6059_8.1481	Positive	763.6059	8.1481	green
M675.5167_8.124	Positive	675.5167	8.124	green
M365.1355_5.7277	Positive	365.1355	5.7277	green
M101.0596_0.3475	Positive	101.0596	0.3475	green
M259.1888_8.1485	Positive	259.1888	8.1485	green
M98.9845_4.4906	Positive	98.9845	4.4906	green
M391.2838_8.1444	Positive	391.2838	8.1444	green

M531.4068_8.9398	Positive	531.4068	8.9398	green
M520.4329_8.2058	Positive	520.4329	8.2058	green
M399.2504_5.813	Positive	399.2504	5.813	green
M149.0593_4.9598	Positive	149.0593	4.9598	green
M429.24_8.1493	Positive	429.24	8.1493	green
M167.0332_8.1441	Positive	167.0332	8.1441	green
M615.4495_8.2613	Positive	615.4495	8.2613	green
M593.4357_8.2733	Positive	593.4357	8.2733	green
M549.4086_8.2991	Positive	549.4086	8.2991	green
M527.3981_8.3268	Positive	527.3981	8.3268	green
M.591.3184_3.0369	Negative	591.3184	3.0369	greenyellow
M.605.3359_3.331	Negative	605.3359	3.331	greenyellow
M.613.302_3.0304	Negative	613.302	3.0304	greenyellow
M.319.1643_2.9542	Negative	319.1643	2.9542	greenyellow
M.673.3225_3.3229	Negative	673.3225	3.3229	greenyellow
M.231.0975_0.289	Negative	231.0975	0.289	greenyellow
M.333.1813_3.4889	Negative	333.1813	3.4889	greenyellow
M593.332_3.0301	Positive	593.332	3.0301	greenyellow
M166.075_0.3976	Positive	166.075	0.3976	greenyellow
M549.2883_8.1134	Positive	549.2883	8.1134	greenyellow
M139.046_0.3867	Positive	139.046	0.3867	greenyellow
M321.1815_2.9517	Positive	321.1815	2.9517	greenyellow
M619.3493_4.4811	Positive	619.3493	4.4811	greenyellow
M316.1391_3.0229	Positive	316.1391	3.0229	greenyellow
M335.1449_4.4093	Positive	335.1449	4.4093	greenyellow
M.603.3198_3.3351	Negative	603.3198	3.3351	un-clustered
M.116.0708_0.3508	Negative	116.0708	0.3508	un-clustered
M.282.0847_0.5037	Negative	282.0847	0.5037	un-clustered
M.587.2888_3.0348	Negative	587.2888	3.0348	un-clustered
M.671.3071_3.3304	Negative	671.3071	3.3304	un-clustered
M.131.0813_0.2861	Negative	131.0813	0.2861	un-clustered
M.283.1635_0.5372	Negative	283.1635	0.5372	un-clustered
M.242.0794_0.303	Negative	242.0794	0.303	un-clustered
M.601.3033_3.3207	Negative	601.3033	3.3207	un-clustered
M.559.3286_3.3284	Negative	559.3286	3.3284	un-clustered
M.182.0266_0.859	Negative	182.0266	0.859	un-clustered
M.324.0225_1.4579	Negative	324.0225	1.4579	un-clustered
M.174.0849_0.3042	Negative	174.0849	0.3042	un-clustered
M.164.0562_0.3582	Negative	164.0562	0.3582	un-clustered
M.184.0714_0.341	Negative	184.0714	0.341	un-clustered
M.432.1725_0.6834	Negative	432.1725	0.6834	un-clustered
M.227.1033_1.4444	Negative	227.1033	1.4444	un-clustered
M.445.187_0.527	Negative	445.187	0.527	un-clustered
M.135.03_0.3767	Negative	135.03	0.3767	un-clustered
M.468.1501_0.682	Negative	468.1501	0.682	un-clustered
M.436.2187_2.1144	Negative	436.2187	2.1144	un-clustered
M.165.0404_0.7521	Negative	165.0404	0.7521	un-clustered

M.255.1328_0.347	Negative	255.1328	0.347	un-clustered
M.288.0778_0.3483	Negative	288.0778	0.3483	un-clustered
M.403.1405_0.3515	Negative	403.1405	0.3515	un-clustered
M.215.139_0.767	Negative	215.139	0.767	un-clustered
M.168.0765_0.2648	Negative	168.0765	0.2648	un-clustered
M.171.0763_0.2836	Negative	171.0763	0.2836	un-clustered
M.161.0917_0.2656	Negative	161.0917	0.2656	un-clustered
M.372.1849_3.5621	Negative	372.1849	3.5621	un-clustered
M.433.1927_0.529	Negative	433.1927	0.529	un-clustered
M.459.2861_2.2262	Negative	459.2861	2.2262	un-clustered
M.291.0423_0.8572	Negative	291.0423	0.8572	un-clustered
M.348.1845_3.5368	Negative	348.1845	3.5368	un-clustered
M.580.2334_0.405	Negative	580.2334	0.405	un-clustered
M288.2893_4.4154	Positive	288.2893	4.4154	un-clustered
M100.0753_0.8499	Positive	100.0753	0.8499	un-clustered
M274.273_4.2483	Positive	274.273	4.2483	un-clustered
M611.2842_5.3914	Positive	611.2842	5.3914	un-clustered
M522.5972_7.9559	Positive	522.5972	7.9559	un-clustered
M437.1926_4.8091	Positive	437.1926	4.8091	un-clustered
M218.2113_2.7599	Positive	218.2113	2.7599	un-clustered
M415.2107_4.8086	Positive	415.2107	4.8086	un-clustered
M316.3204_5.0525	Positive	316.3204	5.0525	un-clustered
M589.3006_3.0315	Positive	589.3006	3.0315	un-clustered
M244.2634_4.4479	Positive	244.2634	4.4479	un-clustered
M185.0912_0.3771	Positive	185.0912	0.3771	un-clustered
M119.0858_4.8086	Positive	119.0858	4.8086	un-clustered
M162.1111_0.2779	Positive	162.1111	0.2779	un-clustered
M256.2637_6.8959	Positive	256.2637	6.8959	un-clustered
M235.18_1.9696	Positive	235.18	1.9696	un-clustered
M494.5656_7.7764	Positive	494.5656	7.7764	un-clustered
M382.258_4.1526	Positive	382.258	4.1526	un-clustered
M149.0224_4.2048	Positive	149.0224	4.2048	un-clustered
M152.0562_0.5034	Positive	152.0562	0.5034	un-clustered
M146.1166_0.291	Positive	146.1166	0.291	un-clustered
M304.2844_4.6169	Positive	304.2844	4.6169	un-clustered
M144.0798_1.3794	Positive	144.0798	1.3794	un-clustered
M207.0496_0.2671	Positive	207.0496	0.2671	un-clustered
M234.2055_2.876	Positive	234.2055	2.876	un-clustered
M284.295_7.5946	Positive	284.295	7.5946	un-clustered
M616.4635_5.6753	Positive	616.4635	5.6753	un-clustered
M230.248_4.2697	Positive	230.248	4.2697	un-clustered
M660.4898_5.6771	Positive	660.4898	5.6771	un-clustered
M104.1063_0.2776	Positive	104.1063	0.2776	un-clustered
M434.1873_0.6811	Positive	434.1873	0.6811	un-clustered
M485.2674_4.4231	Positive	485.2674	4.4231	un-clustered
M572.4383_5.6784	Positive	572.4383	5.6784	un-clustered
M100.0757_0.307	Positive	100.0757	0.307	un-clustered

M137.0456_0.3653	Positive	137.0456	0.3653	un-clustered
M571.4218_8.2851	Positive	571.4218	8.2851	un-clustered
M568.5665_8.5991	Positive	568.5665	8.5991	un-clustered
M159.0758_0.2932	Positive	159.0758	0.2932	un-clustered
M704.5158_5.6688	Positive	704.5158	5.6688	un-clustered
M280.0909_0.276	Positive	280.0909	0.276	un-clustered
M637.46_8.2418	Positive	637.46	8.2418	un-clustered
M748.5426_5.6688	Positive	748.5426	5.6688	un-clustered
M258.1085_0.29	Positive	258.1085	0.29	un-clustered
M379.2372_2.648	Positive	379.2372	2.648	un-clustered
M390.2647_4.545	Positive	390.2647	4.545	un-clustered
M144.0994_0.3073	Positive	144.0994	0.3073	un-clustered
M122.0594_0.8468	Positive	122.0594	0.8468	un-clustered
M230.1845_0.4078	Positive	230.1845	0.4078	un-clustered
M296.0645_0.2756	Positive	296.0645	0.2756	un-clustered
M326.0364_1.4629	Positive	326.0364	1.4629	un-clustered
M528.4115_5.6784	Positive	528.4115	5.6784	un-clustered
M312.3615_6.2537	Positive	312.3615	6.2537	un-clustered
M392.2779_4.8432	Positive	392.2779	4.8432	un-clustered
M540.5378_7.9643	Positive	540.5378	7.9643	un-clustered
M536.6123_8.0429	Positive	536.6123	8.0429	un-clustered
M122.9253_0.264	Positive	122.9253	0.264	un-clustered
M382.2103_4.2707	Positive	382.2103	4.2707	un-clustered
M112.0503_0.3203	Positive	112.0503	0.3203	un-clustered
M216.0135_0.2664	Positive	216.0135	0.2664	un-clustered
M240.1486_2.5023	Positive	240.1486	2.5023	un-clustered
M228.2685_4.5237	Positive	228.2685	4.5237	un-clustered
M121.0632_0.473	Positive	121.0632	0.473	un-clustered
M611.2847_5.1876	Positive	611.2847	5.1876	un-clustered
M268.1537_1.71	Positive	268.1537	1.71	un-clustered
M213.1226_0.4319	Positive	213.1226	0.4319	un-clustered
M466.5338_7.5772	Positive	466.5338	7.5772	un-clustered
M342.2631_4.2272	Positive	342.2631	4.2272	un-clustered
M484.3841_5.6784	Positive	484.3841	5.6784	un-clustered
M792.5678_5.6677	Positive	792.5678	5.6677	un-clustered
M159.1484_0.4809	Positive	159.1484	0.4809	un-clustered
M113.0705_0.2988	Positive	113.0705	0.2988	un-clustered
M596.6586_3.2045	Positive	596.6586	3.2045	un-clustered
M603.3157_3.2513	Positive	603.3157	3.2513	un-clustered
M386.2897_4.3619	Positive	386.2897	4.3619	un-clustered
M158.1167_0.3903	Positive	158.1167	0.3903	un-clustered
M414.2833_4.1527	Positive	414.2833	4.1527	un-clustered
M114.0914_0.3333	Positive	114.0914	0.3333	un-clustered
M128.1064_0.3612	Positive	128.1064	0.3612	un-clustered
M176.1269_0.3373	Positive	176.1269	0.3373	un-clustered
M170.09_0.2576	Positive	170.09	0.2576	un-clustered
M207.1098_1.3021	Positive	207.1098	1.3021	un-clustered

M254.1014_1.09	Positive	254.1014	1.09	un-clustered
M581.9837_3.1861	Positive	581.9837	3.1861	un-clustered
M611.334_3.225	Positive	611.334	3.225	un-clustered
M591.2965_8.123	Positive	591.2965	8.123	un-clustered
M184.0285_0.8467	Positive	184.0285	0.8467	un-clustered
M.497.3349_2.2845	Negative	497.3349	2.2845	grey60
M.487.3075_2.2861	Negative	487.3075	2.2861	grey60
M.514.3254_2.2857	Negative	514.3254	2.2857	grey60
M.451.3294_2.2813	Negative	451.3294	2.2813	grey60
M.565.3249_2.2812	Negative	565.3249	2.2812	grey60
M.112.9857_2.2812	Negative	112.9857	2.2812	grey60
M453.3418_2.2964	Positive	453.3418	2.2964	grey60
M475.3243_2.2894	Positive	475.3243	2.2894	grey60
M213.1739_2.2886	Positive	213.1739	2.2886	grey60
M701.4913_2.6326	Positive	701.4913	2.6326	grey60
M621.3114_8.1199	Positive	621.3114	8.1199	lightcyan
M338.3419_8.2096	Positive	338.3419	8.2096	lightcyan
M666.3701_8.1199	Positive	666.3701	8.1199	lightcyan
M643.2935_8.1197	Positive	643.2935	8.1197	lightcyan
M638.3375_8.1203	Positive	638.3375	8.1203	lightcyan
M761.418_8.1197	Positive	761.418	8.1197	lightcyan
M321.3151_8.2083	Positive	321.3151	8.2083	lightcyan
M659.2661_8.1168	Positive	659.2661	8.1168	lightcyan
M531.2759_7.0585	Positive	531.2759	7.0585	lightcyan
M675.6748_8.2102	Positive	675.6748	8.2102	lightcyan
M199.0573_1.6659	Positive	199.0573	1.6659	lightcyan
M.317.1512_2.8271	Negative	317.1512	2.8271	lightgreen
M.305.1504_2.4501	Negative	305.1504	2.4501	lightgreen
M.587.2873_3.5537	Negative	587.2873	3.5537	lightgreen
M.321.1458_2.0419	Negative	321.1458	2.0419	lightgreen
M.138.0559_1.5796	Negative	138.0559	1.5796	lightgreen
M.259.1449_2.2494	Negative	259.1449	2.2494	lightgreen
M.321.1461_2.2599	Negative	321.1461	2.2599	lightgreen
M180.1017_2.5279	Positive	180.1017	2.5279	lightgreen
M319.1639_2.8192	Positive	319.1639	2.8192	lightgreen
M171.0762_0.2983	Positive	171.0762	0.2983	lightgreen
M482.324_6.493	Positive	482.324	6.493	lightyellow
M619.3497_5.2102	Positive	619.3497	5.2102	lightyellow
M432.2002_2.8698	Positive	432.2002	2.8698	lightyellow
M464.3134_6.4936	Positive	464.3134	6.4936	lightyellow
M167.0848_3.0755	Positive	167.0848	3.0755	lightyellow
M605.3342_5.572	Positive	605.3342	5.572	lightyellow
M504.3078_6.4934	Positive	504.3078	6.4934	lightyellow
M462.2631_5.3339	Positive	462.2631	5.3339	lightyellow
M641.3305_5.2101	Positive	641.3305	5.2101	lightyellow
M466.3273_6.7457	Positive	466.3273	6.7457	lightyellow
M.593.3347_3.0561	Negative	593.3347	3.0561	magenta

M.607.3506_3.3619	Negative	607.3506	3.3619	magenta
M.594.3359_3.0571	Negative	594.3359	3.0571	magenta
M.608.3533_3.3521	Negative	608.3533	3.3521	magenta
M.615.3172_3.0541	Negative	615.3172	3.0541	magenta
M.182.0444_0.5397	Negative	182.0444	0.5397	magenta
M.589.3035_3.5563	Negative	589.3035	3.5563	magenta
M.675.3382_3.3597	Negative	675.3382	3.3597	magenta
M595.3485_3.0533	Positive	595.3485	3.0533	magenta
M596.3501_3.05	Positive	596.3501	3.05	magenta
M155.0807_0.403	Positive	155.0807	0.403	magenta
M141.0648_0.3028	Positive	141.0648	0.3028	magenta
M615.3179_3.0301	Positive	615.3179	3.0301	magenta
M611.3431_2.8949	Positive	611.3431	2.8949	magenta
M617.3334_3.0578	Positive	617.3334	3.0578	magenta
M625.3579_3.208	Positive	625.3579	3.208	magenta
M609.3272_2.8351	Positive	609.3272	2.8351	magenta
M317.1467_3.0518	Positive	317.1467	3.0518	magenta
M.589.303_3.0506	Negative	589.303	3.0506	midnightblue
M.611.2862_3.0479	Negative	611.2862	3.0479	midnightblue
M.591.319_4.3219	Negative	591.319	4.3219	midnightblue
M.79.0582_3.0387	Negative	79.0582	3.0387	midnightblue
M.604.3145_3.177	Negative	604.3145	3.177	midnightblue
M591.3178_3.0401	Positive	591.3178	3.0401	midnightblue
M593.3347_4.3223	Positive	593.3347	4.3223	midnightblue
M613.304_3.0331	Positive	613.304	3.0331	midnightblue
M315.1314_3.0398	Positive	315.1314	3.0398	midnightblue
M303.1696_2.4565	Positive	303.1696	2.4565	midnightblue
M606.3277_3.1689	Positive	606.3277	3.1689	midnightblue
M607.3115_2.8148	Positive	607.3115	2.8148	midnightblue
M.193.034_0.2984	Negative	193.034	0.2984	pink
M.311.1686_5.9839	Negative	311.1686	5.9839	pink
M.261.0161_0.2865	Negative	261.0161	0.2865	pink
M.88.0395_0.3029	Negative	88.0395	0.3029	pink
M.260.9984_0.2954	Negative	260.9984	0.2954	pink
M.113.0241_0.3077	Negative	113.0241	0.3077	pink
M.409.0604_0.3018	Negative	409.0604	0.3018	pink
M.239.0179_0.2995	Negative	239.0179	0.2995	pink
M.331.1296_2.9467	Negative	331.1296	2.9467	pink
M.131.0346_0.3019	Negative	131.0346	0.3019	pink
M.510.2533_3.9927	Negative	510.2533	3.9927	pink
M597.2694_4.7336	Positive	597.2694	4.7336	pink
M611.2851_5.51	Positive	611.2851	5.51	pink
M132.0759_0.3005	Positive	132.0759	0.3005	pink
M114.0659_0.2934	Positive	114.0659	0.2934	pink
M596.2858_4.3339	Positive	596.2858	4.3339	pink
M90.0543_0.2992	Positive	90.0543	0.2992	pink
M143.0807_0.37	Positive	143.0807	0.37	pink

M241.0302_0.3025	Positive	241.0302	0.3025	pink
M633.2976_2.9637	Positive	633.2976	2.9637	pink
M188.1745_0.2603	Positive	188.1745	0.2603	pink
M.96.969_0.3293	Negative	96.969	0.3293	purple
M.78.9594_0.3225	Negative	78.9594	0.3225	purple
M.198.9167_0.3074	Negative	198.9167	0.3074	purple
M.176.9348_0.3164	Negative	176.9348	0.3164	purple
M.216.9271_0.3126	Negative	216.9271	0.3126	purple
M.336.8872_0.3093	Negative	336.8872	0.3093	purple
M.194.9452_0.3144	Negative	194.9452	0.3144	purple
M.254.9256_0.2763	Negative	254.9256	0.2763	purple
M.314.9044_0.3094	Negative	314.9044	0.3094	purple
M.145.0493_1.306	Negative	145.0493	1.306	purple
M.434.8637_0.3047	Negative	434.8637	0.3047	purple
M.412.8809_0.3106	Negative	412.8809	0.3106	purple
M249.1555_1.3332	Positive	249.1555	1.3332	purple
M98.9846_0.3241	Positive	98.9846	0.3241	purple
M227.1742_1.3341	Positive	227.1742	1.3341	purple
M113.9632_0.2468	Positive	113.9632	0.2468	purple
M362.2402_1.8798	Positive	362.2402	1.8798	purple
M.540.3305_5.8069	Negative	540.3305	5.8069	red
M.480.3096_5.8108	Negative	480.3096	5.8108	red
M.608.319_5.8108	Negative	608.319	5.8108	red
M.568.3626_6.377	Negative	568.3626	6.377	red
M.566.3457_5.9833	Negative	566.3457	5.9833	red
M.530.3019_5.8106	Negative	530.3019	5.8106	red
M.639.2954_3.3163	Negative	639.2954	3.3163	red
M.615.3198_5.1325	Negative	615.3198	5.1325	red
M.601.3029_5.4416	Negative	601.3029	5.4416	red
M526.3137_6.7942	Positive	526.3137	6.7942	red
M522.3551_5.9797	Positive	522.3551	5.9797	red
M524.3718_6.3822	Positive	524.3718	6.3822	red
M603.3176_5.4225	Positive	603.3176	5.4225	red
M508.3038_6.7941	Positive	508.3038	6.7941	red
M520.3425_5.5436	Positive	520.3425	5.5436	red
M589.3026_5.5887	Positive	589.3026	5.5887	red
M548.2966_6.7946	Positive	548.2966	6.7946	red
M591.3186_5.759	Positive	591.3186	5.759	red
M341.3035_6.5424	Positive	341.3035	6.5424	red
M603.3189_5.6625	Positive	603.3189	5.6625	red
M603.3169_4.6647	Positive	603.3169	4.6647	red
M470.3256_5.4369	Positive	470.3256	5.4369	red
M583.3188_7.4205	Positive	583.3188	7.4205	red
M184.0728_5.6773	Positive	184.0728	5.6773	red
M494.3242_5.3132	Positive	494.3242	5.3132	red
M544.3381_5.9756	Positive	544.3381	5.9756	red
M496.34_5.804	Positive	496.34	5.804	royalblue

M524.3716_6.5154	Positive	524.3716	6.5154	royalblue
M518.3228_5.8053	Positive	518.3228	5.8053	royalblue
M546.3537_6.5167	Positive	546.3537	6.5167	royalblue
M267.6433_5.8116	Positive	267.6433	5.8116	royalblue
M510.3558_6.1642	Positive	510.3558	6.1642	royalblue
M478.3295_5.8125	Positive	478.3295	5.8125	royalblue
M104.1075_5.8128	Positive	104.1075	5.8128	royalblue
M482.3603_5.9796	Positive	482.3603	5.9796	royalblue
M515.3136_5.8128	Positive	515.3136	5.8128	royalblue
M585.2707_7.8473	Positive	585.2707	7.8473	salmon
M584.2626_7.8363	Positive	584.2626	7.8363	salmon
M299.1388_7.8374	Positive	299.1388	7.8374	salmon
M583.2541_4.201	Positive	583.2541	4.201	salmon
M583.2552_7.8359	Positive	583.2552	7.8359	salmon
M283.1077_7.8356	Positive	283.1077	7.8356	salmon
M585.2707_7.6763	Positive	585.2707	7.6763	salmon
M358.2943_4.7603	Positive	358.2943	4.7603	salmon
M607.2535_7.8535	Positive	607.2535	7.8535	salmon
M299.1394_7.6769	Positive	299.1394	7.6769	salmon
M589.3017_5.8893	Positive	589.3017	5.8893	salmon
M581.2391_7.8381	Positive	581.2391	7.8381	salmon
M75.2126_7.8452	Positive	75.2126	7.8452	salmon
M605.2364_7.8379	Positive	605.2364	7.8379	salmon
M.180.0657_0.4418	Negative	180.0657	0.4418	tan
M.130.0864_0.541	Negative	130.0864	0.541	tan
M.145.0968_0.2662	Negative	145.0968	0.2662	tan
M.383.1222_0.4384	Negative	383.1222	0.4384	tan
M.213.0845_0.2647	Negative	213.0845	0.2647	tan
M.148.0428_0.3876	Negative	148.0428	0.3876	tan
M.163.0393_0.4389	Negative	163.0393	0.4389	tan
M130.0859_0.2607	Positive	130.0859	0.2607	tan
M84.0796_0.2594	Positive	84.0796	0.2594	tan
M133.0318_0.3896	Positive	133.0318	0.3896	tan
M104.0527_0.3889	Positive	104.0527	0.3889	tan
M147.1123_0.2611	Positive	147.1123	0.2611	tan
M150.058_0.3837	Positive	150.058	0.3837	tan
M87.0261_0.3836	Positive	87.0261	0.3836	tan
M.164.0707_0.8702	Negative	164.0707	0.8702	turquoise
M.203.0819_1.396	Negative	203.0819	1.396	turquoise
M.154.0599_0.2741	Negative	154.0599	0.2741	turquoise
M.147.0443_0.8674	Negative	147.0443	0.8674	turquoise
M.351.1323_0.8654	Negative	351.1323	0.8654	turquoise
M.271.0703_1.3966	Negative	271.0703	1.3966	turquoise
M.429.1545_1.3939	Negative	429.1545	1.3939	turquoise
M.173.1027_0.2733	Negative	173.1027	0.2733	turquoise
M.222.0484_0.2628	Negative	222.0484	0.2628	turquoise
M.712.2216_0.8615	Negative	712.2216	0.8615	turquoise

M.322.1478_0.255	Negative	322.1478	0.255	turquoise
M.331.1132_0.2609	Negative	331.1132	0.2609	turquoise
M.241.0903_0.2545	Negative	241.0903	0.2545	turquoise
M120.0806_0.87	Positive	120.0806	0.87	turquoise
M188.0702_1.3973	Positive	188.0702	1.3973	turquoise
M86.096_0.5354	Positive	86.096	0.5354	turquoise
M136.0752_0.44	Positive	136.0752	0.44	turquoise
M123.0442_0.4369	Positive	123.0442	0.4369	turquoise
M146.0599_1.3963	Positive	146.0599	1.3963	turquoise
M165.054_0.4388	Positive	165.054	0.4388	turquoise
M72.0808_0.3471	Positive	72.0808	0.3471	turquoise
M103.0543_0.8669	Positive	103.0543	0.8669	turquoise
M119.0489_0.4383	Positive	119.0489	0.4383	turquoise
M166.0862_0.8662	Positive	166.0862	0.8662	turquoise
M147.044_0.4378	Positive	147.044	0.4378	turquoise
M132.1013_0.5355	Positive	132.1013	0.5355	turquoise
M116.0696_0.3031	Positive	116.0696	0.3031	turquoise
M175.1184_0.2707	Positive	175.1184	0.2707	turquoise
M70.0652_0.3021	Positive	70.0652	0.3021	turquoise
M110.0705_0.2626	Positive	110.0705	0.2626	turquoise
M182.0806_0.4378	Positive	182.0806	0.4378	turquoise
M69.0701_0.5033	Positive	69.0701	0.5033	turquoise
M120.0807_1.6347	Positive	120.0807	1.6347	turquoise
M131.0507_0.8626	Positive	131.0507	0.8626	turquoise
M95.0492_0.4377	Positive	95.0492	0.4377	turquoise
M91.0539_0.437	Positive	91.0539	0.437	turquoise
M107.0494_0.8651	Positive	107.0494	0.8651	turquoise
M156.0759_0.259	Positive	156.0759	0.259	turquoise
M159.0914_1.396	Positive	159.0914	1.396	turquoise
M79.0545_0.8643	Positive	79.0545	0.8643	turquoise
M132.0806_1.3949	Positive	132.0806	1.3949	turquoise
M93.0695_0.8642	Positive	93.0695	0.8642	turquoise
M149.0613_0.8616	Positive	149.0613	0.8616	turquoise
M223.0239_0.266	Positive	223.0239	0.266	turquoise
M205.0981_1.395	Positive	205.0981	1.395	turquoise
M118.0652_1.3923	Positive	118.0652	1.3923	turquoise
M129.066_0.2997	Positive	129.066	0.2997	turquoise
M136.0738_1.2046	Positive	136.0738	1.2046	turquoise
M83.0596_0.3471	Positive	83.0596	0.3471	turquoise
M447.2015_0.5293	Positive	447.2015	0.5293	turquoise
M178.0574_0.2759	Positive	178.0574	0.2759	turquoise
M188.0689_0.8615	Positive	188.0689	0.8615	turquoise
M189.1579_0.2674	Positive	189.1579	0.2674	turquoise
M.455.2471_4.939	Negative	455.2471	4.939	yellow
M.611.2878_5.5846	Negative	611.2878	5.5846	yellow
M.597.2744_5.9533	Negative	597.2744	5.9533	yellow
M.471.2432_4.2884	Negative	471.2432	4.2884	yellow

M.453.2313_4.7124	Negative	453.2313	4.7124	yellow
M.512.2684_4.2828	Negative	512.2684	4.2828	yellow
M.633.2701_5.5599	Negative	633.2701	5.5599	yellow
M.477.229_4.9363	Negative	477.229	4.9363	yellow
M613.3015_5.5823	Positive	613.3015	5.5823	yellow
M599.2869_5.9485	Positive	599.2869	5.9485	yellow
M598.3006_5.4799	Positive	598.3006	5.4799	yellow
M577.2807_7.5333	Positive	577.2807	7.5333	yellow
M615.3181_4.4521	Positive	615.3181	4.4521	yellow
M635.2846_5.5894	Positive	635.2846	5.5894	yellow
M736.4545_7.4082	Positive	736.4545	7.4082	yellow
M615.3177_4.6006	Positive	615.3177	4.6006	yellow
M583.3029_5.9727	Positive	583.3029	5.9727	yellow
M357.2782_4.2867	Positive	357.2782	4.2867	yellow
M775.3188_4.4819	Positive	775.3188	4.4819	yellow
M584.2864_7.012	Positive	584.2864	7.012	yellow
M282.2783_7.0525	Positive	282.2783	7.0525	yellow
M734.4449_7.4067	Positive	734.4449	7.4067	yellow
M510.1703_4.9321	Positive	510.1703	4.9321	yellow
M582.2967_5.9731	Positive	582.2967	5.9731	yellow
M620.2866_5.478	Positive	620.2866	5.478	yellow
M621.2696_5.932	Positive	621.2696	5.932	yellow
M384.2207_4.5541	Positive	384.2207	4.5541	yellow
M597.2924_5.4718	Positive	597.2924	5.4718	yellow
M508.2421_4.6505	Positive	508.2421	4.6505	yellow
M313.154_3.1441	Positive	313.154	3.1441	yellow
M679.4347_7.4504	Positive	679.4347	7.4504	yellow
M144.0806_1.5359	Positive	144.0806	1.5359	yellow
M629.2955_5.0678	Positive	629.2955	5.0678	yellow
M750.4729_7.6158	Positive	750.4729	7.6158	yellow

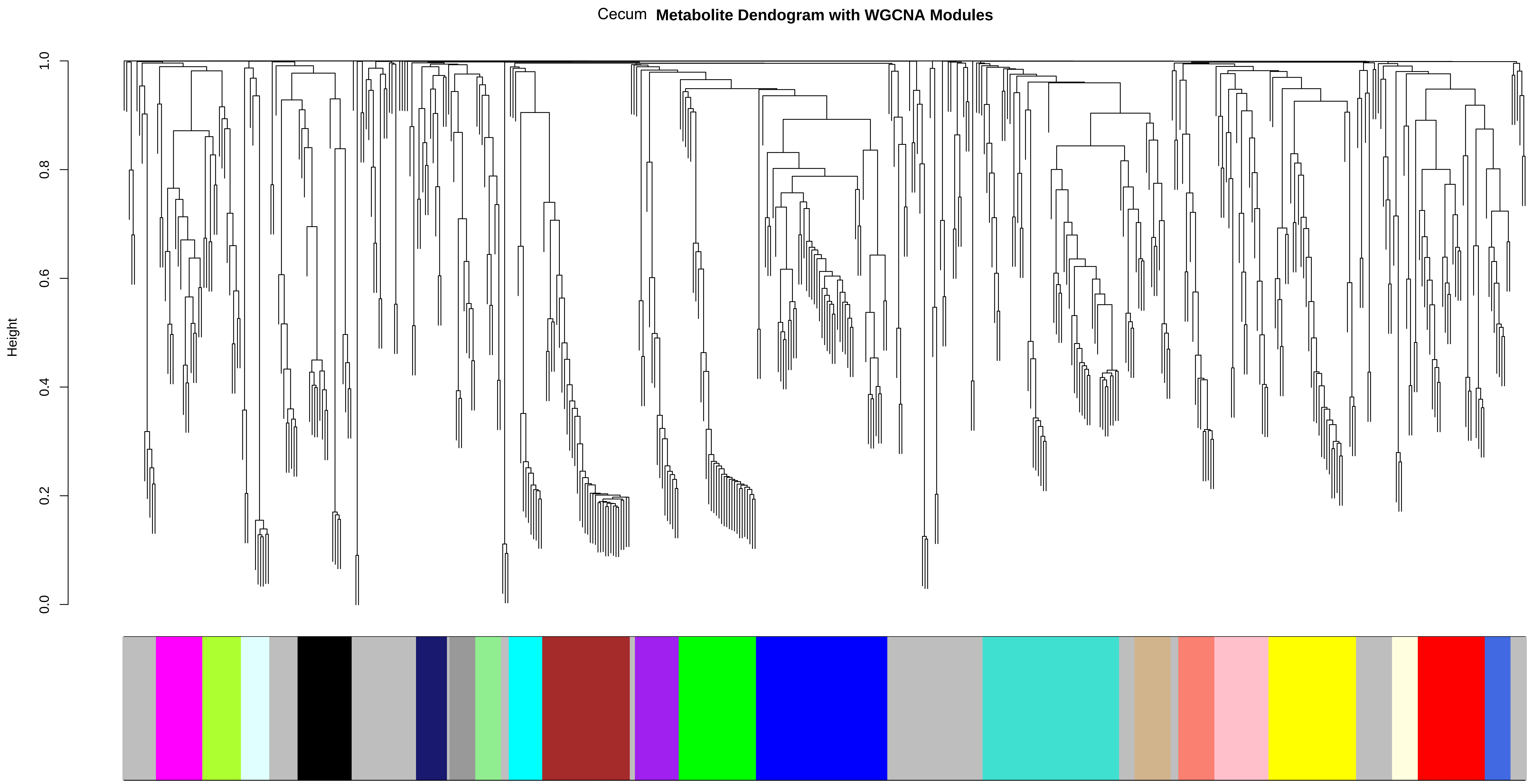

Cecum Metabolite Dendogram with WGCNA Modules
Height
1.0
0.8
0.6
0.4
0.2
0.0

Sigmoid Metabolites				
Metabolite	Dection Ion Mode	Mass (m/z)	Retention Time (Minutes)	Module
M-112.9851_0.2746	Negative	112.9851	0.2746	black
M-248.96_0.2722	Negative	248.96	0.2722	black
M-316.9476_0.2681	Negative	316.9476	0.2681	black
M-180.9723_0.2656	Negative	180.9723	0.2656	black
M-384.9348_0.2676	Negative	384.9348	0.2676	black
M-452.9229_0.2677	Negative	452.9229	0.2677	black
M-110.9753_0.3347	Negative	110.9753	0.3347	black
M-588.8979_0.2663	Negative	588.8979	0.2663	black
M-520.9088_0.2663	Negative	520.9088	0.2663	black
M-154.9735_0.265	Negative	154.9735	0.265	black
M-656.8846_0.2663	Negative	656.8846	0.2663	black
M-89.0242_0.3893	Negative	89.0242	0.3893	black
M475.324_2.2783	Positive	475.324	2.2783	black
M90.9772_0.2743	Positive	90.9772	0.2743	black
M226.9524_0.2763	Positive	226.9524	0.2763	black
M188.1274_0.3879	Positive	188.1274	0.3879	black
M217.1032_1.1884	Positive	217.1032	1.1884	black
M172.0962_0.3294	Positive	172.0962	0.3294	black
M132.0045_0.2534	Positive	132.0045	0.2534	black
M174.1119_0.3378	Positive	174.1119	0.3378	black
M-96.969_0.321	Negative	96.969	0.321	blue
M-194.9454_0.3209	Negative	194.9454	0.3209	blue
M-292.9225_0.3223	Negative	292.9225	0.3223	blue
M-176.9351_0.3252	Negative	176.9351	0.3252	blue
M-78.9595_0.3222	Negative	78.9595	0.3222	blue
M-390.8995_0.3184	Negative	390.8995	0.3184	blue
M-488.878_0.3203	Negative	488.878	0.3203	blue
M-586.8552_0.3219	Negative	586.8552	0.3219	blue
M-684.832_0.3176	Negative	684.832	0.3176	blue
M-510.859_0.2918	Negative	510.859	0.2918	blue
M-158.9246_0.3191	Negative	158.9246	0.3191	blue
M-782.8098_0.322	Negative	782.8098	0.322	blue
M-434.8629_0.2776	Negative	434.8629	0.2776	blue
M-728.7928_0.2873	Negative	728.7928	0.2873	blue
M102.1272_0.384	Positive	102.1272	0.384	blue
M130.1585_1.6648	Positive	130.1585	1.6648	blue
M199.0573_1.6596	Positive	199.0573	1.6596	blue
M239.0881_2.7716	Positive	239.0881	2.7716	blue
M114.091_1.449	Positive	114.091	1.449	blue
M116.0126_0.3075	Positive	116.0126	0.3075	blue
M196.9621_0.3149	Positive	196.9621	0.3149	blue
M118.123_0.328	Positive	118.123	0.328	blue
M183.0622_1.381	Positive	183.0622	1.381	blue
M97.0643_2.7708	Positive	97.0643	2.7708	blue
M294.9394_0.3069	Positive	294.9394	0.3069	blue

M392.9164_0.3072	Positive	392.9164	0.3072	blue
M100.0753_1.0563	Positive	100.0753	1.0563	blue
M270.1485_2.9599	Positive	270.1485	2.9599	blue
M507.9208_0.305	Positive	507.9208	0.305	blue
M157.0853_2.7696	Positive	157.0853	2.7696	blue
M125.0583_1.6227	Positive	125.0583	1.6227	blue
M409.9426_0.3071	Positive	409.9426	0.3071	blue
M213.9895_0.3119	Positive	213.9895	0.3119	blue
M490.8932_0.3113	Positive	490.8932	0.3113	blue
M100.1115_0.7426	Positive	100.1115	0.7426	blue
M588.8707_0.3065	Positive	588.8707	0.3065	blue
M311.9656_0.3034	Positive	311.9656	0.3034	blue
M292.023_0.6357	Positive	292.023	0.6357	blue
M117.0543_1.6556	Positive	117.0543	1.6556	blue
M98.9837_0.6315	Positive	98.9837	0.6315	blue
M703.8754_0.3058	Positive	703.8754	0.3058	blue
M605.898_0.3028	Positive	605.898	0.3028	blue
M83.0849_0.7283	Positive	83.0849	0.7283	blue
M577.3593_2.9628	Positive	577.3593	2.9628	brown
M621.3863_3.0325	Positive	621.3863	3.0325	brown
M557.8243_2.9333	Positive	557.8243	2.9333	brown
M555.3468_2.9321	Positive	555.3468	2.9321	brown
M535.811_2.895	Positive	535.811	2.895	brown
M533.3332_2.8943	Positive	533.3332	2.8943	brown
M513.7973_2.8463	Positive	513.7973	2.8463	brown
M623.8635_3.049	Positive	623.8635	3.049	brown
M643.3996_3.061	Positive	643.3996	3.061	brown
M511.3195_2.8512	Positive	511.3195	2.8512	brown
M491.7839_2.8079	Positive	491.7839	2.8079	brown
M480.794_2.8076	Positive	480.794	2.8076	brown
M665.4109_3.085	Positive	665.4109	3.085	brown
M502.8065_2.8447	Positive	502.8065	2.8447	brown
M489.3066_2.807	Positive	489.3066	2.807	brown
M524.8198_2.8997	Positive	524.8198	2.8997	brown
M687.4236_3.114	Positive	687.4236	3.114	brown
M469.7703_2.765	Positive	469.7703	2.765	brown
M458.7806_2.7594	Positive	458.7806	2.7594	brown
M436.7686_2.7257	Positive	436.7686	2.7257	brown
M546.8327_2.9321	Positive	546.8327	2.9321	brown
M709.4385_3.1467	Positive	709.4385	3.1467	brown
M439.2462_2.7261	Positive	439.2462	2.7261	brown
M417.232_2.674	Positive	417.232	2.674	brown
M612.8709_3.0322	Positive	612.8709	3.0322	brown
M538.2965_2.9101	Positive	538.2965	2.9101	brown
M784.4861_2.6207	Positive	784.4861	2.6207	brown
M560.3034_2.931	Positive	560.3034	2.931	brown
M450.2376_2.726	Positive	450.2376	2.726	brown

M447.7574_2.724	Positive	447.7574	2.724	brown
M467.2906_2.7625	Positive	467.2906	2.7625	brown
M414.7535_2.6752	Positive	414.7535	2.6752	brown
M494.2901_2.8578	Positive	494.2901	2.8578	brown
M516.3043_2.8993	Positive	516.3043	2.8993	brown
M-603.3187_3.3277	Negative	603.3187	3.3277	cyan
M-611.2853_3.0441	Negative	611.2853	3.0441	cyan
M-671.3072_3.323	Negative	671.3072	3.323	cyan
M-324.0232_1.455	Negative	324.0232	1.455	cyan
M-559.3278_3.3218	Negative	559.3278	3.3218	cyan
M-305.1503_2.4579	Negative	305.1503	2.4579	cyan
M-639.2945_3.321	Negative	639.2945	3.321	cyan
M-432.1725_0.6656	Negative	432.1725	0.6656	cyan
M617.3324_4.4313	Positive	617.3324	4.4313	cyan
M326.0363_1.4553	Positive	326.0363	1.4553	cyan
M254.1014_1.0605	Positive	254.1014	1.0605	cyan
M317.1843_2.9316	Positive	317.1843	2.9316	cyan
M-314.9055_0.289	Negative	314.9055	0.289	green
M-216.928_0.2831	Negative	216.928	0.2831	green
M-412.8816_0.2926	Negative	412.8816	0.2926	green
M-198.9176_0.2768	Negative	198.9176	0.2768	green
M-336.8868_0.2805	Negative	336.8868	0.2805	green
M-608.8351_0.2948	Negative	608.8351	0.2948	green
M-456.8444_0.2784	Negative	456.8444	0.2784	green
M-532.8398_0.2894	Negative	532.8398	0.2894	green
M-318.8763_0.2771	Negative	318.8763	0.2771	green
M-706.8124_0.2929	Negative	706.8124	0.2929	green
M-630.8133_0.289	Negative	630.8133	0.289	green
M-576.8039_0.2775	Negative	576.8039	0.2775	green
M-554.8214_0.2823	Negative	554.8214	0.2823	green
M-254.9258_0.2715	Negative	254.9258	0.2715	green
M120.9675_0.2939	Positive	120.9675	0.2939	green
M218.9442_0.2946	Positive	218.9442	0.2946	green
M142.9498_0.2831	Positive	142.9498	0.2831	green
M414.8986_0.2882	Positive	414.8986	0.2882	green
M240.9267_0.2818	Positive	240.9267	0.2818	green
M164.9313_0.2728	Positive	164.9313	0.2728	green
M262.9086_0.284	Positive	262.9086	0.284	green
M192.1609_0.3527	Positive	192.1609	0.3527	green
M338.9027_0.2869	Positive	338.9027	0.2869	green
M382.8668_0.2775	Positive	382.8668	0.2775	green
M436.8812_0.2825	Positive	436.8812	0.2825	green
M360.8848_0.2805	Positive	360.8848	0.2805	green
M-445.1995_2.75	Negative	445.1995	2.75	greenyellow
M-540.3302_5.8143	Negative	540.3302	5.8143	greenyellow
M-242.0791_0.3079	Negative	242.0791	0.3079	greenyellow
M496.3385_5.8089	Positive	496.3385	5.8089	greenyellow

M447.2124_2.7503	Positive	447.2124	2.7503	greenyellow
M104.1071_0.2777	Positive	104.1071	0.2777	greenyellow
M167.0852_3.058	Positive	167.0852	3.058	greenyellow
M432.1992_2.8607	Positive	432.1992	2.8607	greenyellow
M258.1121_0.2956	Positive	258.1121	0.2956	greenyellow
M296.0667_0.2762	Positive	296.0667	0.2762	greenyellow
M280.0935_0.2802	Positive	280.0935	0.2802	greenyellow
M158.1167_0.3949	Positive	158.1167	0.3949	greenyellow
M518.3221_5.8101	Positive	518.3221	5.8101	greenyellow
M591.2958_8.1268	Positive	591.2958	8.1268	greenyellow
M176.1286_0.3459	Positive	176.1286	0.3459	greenyellow
M-242.1757_3.5522	Negative	242.1757	3.5522	un-clustered
M-194.0815_4.7284	Negative	194.0815	4.7284	un-clustered
M-213.0143_3.0981	Negative	213.0143	3.0981	un-clustered
M-589.3037_3.0471	Negative	589.3037	3.0471	un-clustered
M-497.3341_2.2811	Negative	497.3341	2.2811	un-clustered
M-605.3348_3.3185	Negative	605.3348	3.3185	un-clustered
M-591.3203_3.0304	Negative	591.3203	3.0304	un-clustered
M-487.3048_2.2819	Negative	487.3048	2.2819	un-clustered
M-145.0968_0.2642	Negative	145.0968	0.2642	un-clustered
M-514.324_2.2809	Negative	514.324	2.2809	un-clustered
M-213.0843_0.2653	Negative	213.0843	0.2653	un-clustered
M-68.9971_0.4077	Negative	68.9971	0.4077	un-clustered
M-131.0815_0.2923	Negative	131.0815	0.2923	un-clustered
M-112.9853_0.4082	Negative	112.9853	0.4082	un-clustered
M-330.8779_0.2826	Negative	330.8779	0.2826	un-clustered
M-304.9139_0.2537	Negative	304.9139	0.2537	un-clustered
M-227.1034_1.4408	Negative	227.1034	1.4408	un-clustered
M-130.9657_0.2548	Negative	130.9657	0.2548	un-clustered
M-587.2885_3.0094	Negative	587.2885	3.0094	un-clustered
M-383.1814_2.1754	Negative	383.1814	2.1754	un-clustered
M-617.334_3.6584	Negative	617.334	3.6584	un-clustered
M-145.0495_1.3091	Negative	145.0495	1.3091	un-clustered
M-428.8518_0.2798	Negative	428.8518	0.2798	un-clustered
M-601.3035_3.2988	Negative	601.3035	3.2988	un-clustered
M-317.1503_2.8089	Negative	317.1503	2.8089	un-clustered
M-275.0549_0.247	Negative	275.0549	0.247	un-clustered
M-450.8341_0.2807	Negative	450.8341	0.2807	un-clustered
M-174.088_0.2977	Negative	174.088	0.2977	un-clustered
M-232.9025_0.2859	Negative	232.9025	0.2859	un-clustered
M-139.0059_0.5129	Negative	139.0059	0.5129	un-clustered
M-352.8623_0.2837	Negative	352.8623	0.2837	un-clustered
M-619.3474_3.6896	Negative	619.3474	3.6896	un-clustered
M-472.8195_0.2759	Negative	472.8195	0.2759	un-clustered
M-134.0459_0.3321	Negative	134.0459	0.3321	un-clustered
M-613.3011_3.0293	Negative	613.3011	3.0293	un-clustered
M-673.3209_3.3132	Negative	673.3209	3.3132	un-clustered

M-193.0347_0.2935	Negative	193.0347	0.2935	un-clustered
M-334.8555_0.289	Negative	334.8555	0.289	un-clustered
M288.2889_4.3996	Positive	288.2889	4.3996	un-clustered
M591.3165_3.0365	Positive	591.3165	3.0365	un-clustered
M100.0752_0.844	Positive	100.0752	0.844	un-clustered
M274.2731_4.2277	Positive	274.2731	4.2277	un-clustered
M593.3309_3.0247	Positive	593.3309	3.0247	un-clustered
M227.1744_1.3254	Positive	227.1744	1.3254	un-clustered
M338.3417_8.2108	Positive	338.3417	8.2108	un-clustered
M315.1469_1.3011	Positive	315.1469	1.3011	un-clustered
M155.0809_0.3985	Positive	155.0809	0.3985	un-clustered
M522.597_7.9391	Positive	522.597	7.9391	un-clustered
M595.3469_3.0469	Positive	595.3469	3.0469	un-clustered
M98.985_0.3349	Positive	98.985	0.3349	un-clustered
M249.1559_1.3248	Positive	249.1559	1.3248	un-clustered
M218.2107_2.7429	Positive	218.2107	2.7429	un-clustered
M268.1898_2.2693	Positive	268.1898	2.2693	un-clustered
M453.3418_2.2794	Positive	453.3418	2.2794	un-clustered
M86.0601_0.5164	Positive	86.0601	0.5164	un-clustered
M316.3201_5.0396	Positive	316.3201	5.0396	un-clustered
M149.0231_4.2133	Positive	149.0231	4.2133	un-clustered
M611.2859_5.3949	Positive	611.2859	5.3949	un-clustered
M98.9843_4.4893	Positive	98.9843	4.4893	un-clustered
M101.0597_0.3673	Positive	101.0597	0.3673	un-clustered
M235.1792_1.9584	Positive	235.1792	1.9584	un-clustered
M596.3475_3.04	Positive	596.3475	3.04	un-clustered
M524.3693_6.5163	Positive	524.3693	6.5163	un-clustered
M589.3009_2.9972	Positive	589.3009	2.9972	un-clustered
M185.0913_0.391	Positive	185.0913	0.391	un-clustered
M130.0866_0.2631	Positive	130.0866	0.2631	un-clustered
M244.263_4.4274	Positive	244.263	4.4274	un-clustered
M273.1666_2.4083	Positive	273.1666	2.4083	un-clustered
M437.1925_4.8087	Positive	437.1925	4.8087	un-clustered
M415.21_4.8087	Positive	415.21	4.8087	un-clustered
M84.9563_0.2493	Positive	84.9563	0.2493	un-clustered
M84.0803_0.2594	Positive	84.0803	0.2594	un-clustered
M146.1173_0.2882	Positive	146.1173	0.2882	un-clustered
M174.992_4.4893	Positive	174.992	4.4893	un-clustered
M494.564_7.7545	Positive	494.564	7.7545	un-clustered
M118.0862_0.3312	Positive	118.0862	0.3312	un-clustered
M139.9888_8.0477	Positive	139.9888	8.0477	un-clustered
M132.0773_0.3037	Positive	132.0773	0.3037	un-clustered
M152.0563_0.4676	Positive	152.0563	0.4676	un-clustered
M116.0706_0.2886	Positive	116.0706	0.2886	un-clustered
M282.2787_5.0784	Positive	282.2787	5.0784	un-clustered
M637.3048_7.1635	Positive	637.3048	7.1635	un-clustered
M162.1127_0.2784	Positive	162.1127	0.2784	un-clustered

M348.9872_4.4892	Positive	348.9872	4.4892	un-clustered
M256.2633_6.8978	Positive	256.2633	6.8978	un-clustered
M350.9866_4.4904	Positive	350.9866	4.4904	un-clustered
M482.3242_6.4931	Positive	482.3242	6.4931	un-clustered
M385.195_2.1639	Positive	385.195	2.1639	un-clustered
M160.1328_0.3847	Positive	160.1328	0.3847	un-clustered
M100.0761_0.3093	Positive	100.0761	0.3093	un-clustered
M209.1643_1.3247	Positive	209.1643	1.3247	un-clustered
M508.5839_7.9121	Positive	508.5839	7.9121	un-clustered
M70.0656_0.296	Positive	70.0656	0.296	un-clustered
M141.0659_0.3013	Positive	141.0659	0.3013	un-clustered
M119.0851_4.8123	Positive	119.0851	4.8123	un-clustered
M250.9993_4.4886	Positive	250.9993	4.4886	un-clustered
M434.1867_0.6621	Positive	434.1867	0.6621	un-clustered
M114.0668_0.2851	Positive	114.0668	0.2851	un-clustered
M191.0769_0.2635	Positive	191.0769	0.2635	un-clustered
M621.311_8.1152	Positive	621.311	8.1152	un-clustered
M245.1348_2.0142	Positive	245.1348	2.0142	un-clustered
M613.3013_5.5673	Positive	613.3013	5.5673	un-clustered
M379.2363_2.6316	Positive	379.2363	2.6316	un-clustered
M526.3129_6.8492	Positive	526.3129	6.8492	un-clustered
M124.0395_0.3735	Positive	124.0395	0.3735	un-clustered
M332.8854_0.3243	Positive	332.8854	0.3243	un-clustered
M613.3071_3.0358	Positive	613.3071	3.0358	un-clustered
M331.209_2.8322	Positive	331.209	2.8322	un-clustered
M619.3535_4.4699	Positive	619.3535	4.4699	un-clustered
M137.0461_0.3708	Positive	137.0461	0.3708	un-clustered
M144.079_1.3518	Positive	144.079	1.3518	un-clustered
M597.269_4.7068	Positive	597.269	4.7068	un-clustered
M480.5502_7.7237	Positive	480.5502	7.7237	un-clustered
M321.3135_8.2099	Positive	321.3135	8.2099	un-clustered
M445.8266_0.342	Positive	445.8266	0.342	un-clustered
M125.9868_0.2435	Positive	125.9868	0.2435	un-clustered
M475.3238_1.3248	Positive	475.3238	1.3248	un-clustered
M585.2705_7.8413	Positive	585.2705	7.8413	un-clustered
M207.0509_0.2655	Positive	207.0509	0.2655	un-clustered
M536.6115_8.0891	Positive	536.6115	8.0891	un-clustered
M214.9176_0.2446	Positive	214.9176	0.2446	un-clustered
M230.1846_0.4039	Positive	230.1846	0.4039	un-clustered
M150.0576_0.3934	Positive	150.0576	0.3934	un-clustered
M603.3162_3.2391	Positive	603.3162	3.2391	un-clustered
M112.0506_0.3352	Positive	112.0506	0.3352	un-clustered
M90.055_0.3017	Positive	90.055	0.3017	un-clustered
M362.2404_1.8712	Positive	362.2404	1.8712	un-clustered
M347.8494_0.3422	Positive	347.8494	0.3422	un-clustered
M654.3316_7.1632	Positive	654.3316	7.1632	un-clustered
M147.1129_0.2619	Positive	147.1129	0.2619	un-clustered

M371.1796_2.0884	Positive	371.1796	2.0884	un-clustered
M230.248_4.2518	Positive	230.248	4.2518	un-clustered
M643.2921_8.122	Positive	643.2921	8.122	un-clustered
M776.2319_8.7894	Positive	776.2319	8.7894	un-clustered
M102.0541_0.3917	Positive	102.0541	0.3917	un-clustered
M302.3051_5.2192	Positive	302.3051	5.2192	un-clustered
M666.3702_8.1182	Positive	666.3702	8.1182	un-clustered
M454.2894_5.7892	Positive	454.2894	5.7892	un-clustered
M86.0962_1.959	Positive	86.0962	1.959	un-clustered
M159.0767_0.2953	Positive	159.0767	0.2953	un-clustered
M702.2124_8.4252	Positive	702.2124	8.4252	un-clustered
M430.8618_0.2929	Positive	430.8618	0.2929	un-clustered
M240.2314_4.2929	Positive	240.2314	4.2929	un-clustered
M154.0825_2.1583	Positive	154.0825	2.1583	un-clustered
M223.0257_0.2673	Positive	223.0257	0.2673	un-clustered
M390.2017_2.1516	Positive	390.2017	2.1516	un-clustered
M171.0766_0.3068	Positive	171.0766	0.3068	un-clustered
M181.072_1.3527	Positive	181.072	1.3527	un-clustered
M200.0394_0.2696	Positive	200.0394	0.2696	un-clustered
M299.1389_7.8379	Positive	299.1389	7.8379	un-clustered
M372.2428_4.5395	Positive	372.2428	4.5395	un-clustered
M234.2053_2.8572	Positive	234.2053	2.8572	un-clustered
M129.1343_0.2449	Positive	129.1343	0.2449	un-clustered
M128.107_0.377	Positive	128.107	0.377	un-clustered
M144.0802_1.7079	Positive	144.0802	1.7079	un-clustered
M159.1482_0.4685	Positive	159.1482	0.4685	un-clustered
M158.0952_1.7886	Positive	158.0952	1.7886	un-clustered
M303.1687_2.4854	Positive	303.1687	2.4854	un-clustered
M312.3621_6.2213	Positive	312.3621	6.2213	un-clustered
M114.0917_0.317	Positive	114.0917	0.317	un-clustered
M121.0611_0.461	Positive	121.0611	0.461	un-clustered
M256.8925_0.283	Positive	256.8925	0.283	un-clustered
M615.3166_4.4598	Positive	615.3166	4.4598	un-clustered
M736.4555_7.405	Positive	736.4555	7.405	un-clustered
M145.1341_0.3663	Positive	145.1341	0.3663	un-clustered
M174.1848_2.751	Positive	174.1848	2.751	un-clustered
M750.4712_7.6099	Positive	750.4712	7.6099	un-clustered
M122.9265_0.2686	Positive	122.9265	0.2686	un-clustered
M615.3154_3.0227	Positive	615.3154	3.0227	un-clustered
M466.5344_7.5438	Positive	466.5344	7.5438	un-clustered
M393.297_8.1561	Positive	393.297	8.1561	magenta
M550.6313_8.1126	Positive	550.6313	8.1126	magenta
M413.2663_8.1526	Positive	413.2663	8.1526	magenta
M459.3018_8.254	Positive	459.3018	8.254	magenta
M337.1039_4.9636	Positive	337.1039	4.9636	magenta
M149.0229_8.1503	Positive	149.0229	8.1503	magenta
M783.574_8.1498	Positive	783.574	8.1498	magenta

M149.0233_5.9553	Positive	149.0233	5.9553	magenta
M504.3595_8.2537	Positive	504.3595	8.2537	magenta
M371.3149_8.1514	Positive	371.3149	8.1514	magenta
M129.0552_8.1518	Positive	129.0552	8.1518	magenta
M301.1404_5.9553	Positive	301.1404	5.9553	magenta
M763.6055_8.15	Positive	763.6055	8.15	magenta
M675.5161_8.1222	Positive	675.5161	8.1222	magenta
M365.1351_5.7347	Positive	365.1351	5.7347	magenta
M610.1829_8.8144	Positive	610.1829	8.8144	magenta
M259.1894_8.1513	Positive	259.1894	8.1513	magenta
M147.0628_8.14	Positive	147.0628	8.14	magenta
M564.3565_2.2921	Positive	564.3565	2.2921	midnightblue
M608.382_2.3719	Positive	608.382	2.3719	midnightblue
M569.3125_2.2928	Positive	569.3125	2.2928	midnightblue
M652.4092_2.4465	Positive	652.4092	2.4465	midnightblue
M613.3386_2.3699	Positive	613.3386	2.3699	midnightblue
M696.4347_2.5002	Positive	696.4347	2.5002	midnightblue
M657.3643_2.4447	Positive	657.3643	2.4447	midnightblue
M740.4621_2.5724	Positive	740.4621	2.5724	midnightblue
M547.3283_2.287	Positive	547.3283	2.287	midnightblue
M428.2244_2.6762	Positive	428.2244	2.6762	midnightblue
M701.3915_2.501	Positive	701.3915	2.501	midnightblue
M596.6587_3.1998	Positive	596.6587	3.1998	pink
M609.6818_3.2132	Positive	609.6818	3.2132	pink
M327.1903_2.1995	Positive	327.1903	2.1995	pink
M611.3327_3.2208	Positive	611.3327	3.2208	pink
M595.007_3.1999	Positive	595.007	3.1999	pink
M581.9832_3.1714	Positive	581.9832	3.1714	pink
M626.0076_3.2351	Positive	626.0076	3.2351	pink
M624.3556_3.2398	Positive	624.3556	3.2398	pink
M567.3072_3.1485	Positive	567.3072	3.1485	pink
M580.3275_3.1785	Positive	580.3275	3.1785	pink
M640.684_3.2579	Positive	640.684	3.2579	pink
M639.0323_3.255	Positive	639.0323	3.255	pink
M565.6542_3.1515	Positive	565.6542	3.1515	pink
M552.6323_3.1246	Positive	552.6323	3.1246	pink
M550.9806_3.1287	Positive	550.9806	3.1287	pink
M341.2052_2.3342	Positive	341.2052	2.3342	pink
M537.9532_3.101	Positive	537.9532	3.101	pink
M597.3297_2.8482	Positive	597.3297	2.8482	pink
M627.6774_3.2639	Positive	627.6774	3.2639	pink
M619.34_2.8681	Positive	619.34	2.8681	pink
M-164.0707_0.8612	Negative	164.0707	0.8612	purple
M-147.0444_0.858	Negative	147.0444	0.858	purple
M-351.1321_0.8582	Negative	351.1321	0.8582	purple
M-712.2202_0.8531	Negative	712.2202	0.8531	purple
M-547.1384_0.8522	Negative	547.1384	0.8522	purple

M120.0805_0.8526	Positive	120.0805	0.8526	purple
M103.0541_0.8512	Positive	103.0541	0.8512	purple
M166.0857_0.8485	Positive	166.0857	0.8485	purple
M120.0806_1.6236	Positive	120.0806	1.6236	purple
M110.0712_0.2614	Positive	110.0712	0.2614	purple
M131.051_0.8491	Positive	131.051	0.8491	purple
M107.0492_0.8483	Positive	107.0492	0.8483	purple
M79.0543_0.844	Positive	79.0543	0.844	purple
M156.0764_0.2596	Positive	156.0764	0.2596	purple
M149.0618_0.846	Positive	149.0618	0.846	purple
M93.0696_0.8451	Positive	93.0696	0.8451	purple
M-174.9549_0.2531	Negative	174.9549	0.2531	red
M-119.0341_0.3838	Negative	119.0341	0.3838	red
M-128.0347_0.4221	Negative	128.0347	0.4221	red
M224.0914_3.1824	Positive	224.0914	3.1824	red
M72.0809_0.3195	Positive	72.0809	0.3195	red
M263.1377_1.654	Positive	263.1377	1.654	red
M238.1041_1.9697	Positive	238.1041	1.9697	red
M295.1638_2.1241	Positive	295.1638	2.1241	red
M224.091_2.7617	Positive	224.091	2.7617	red
M229.0682_7.9763	Positive	229.0682	7.9763	red
M249.1564_1.5617	Positive	249.1564	1.5617	red
M126.0909_0.3938	Positive	126.0909	0.3938	red
M227.1742_1.5617	Positive	227.1742	1.5617	red
M267.0834_2.6441	Positive	267.0834	2.6441	red
M219.1088_1.5333	Positive	219.1088	1.5333	red
M144.101_0.3104	Positive	144.101	0.3104	red
M179.1173_1.5343	Positive	179.1173	1.5343	red
M224.1208_1.2448	Positive	224.1208	1.2448	red
M222.1479_1.3951	Positive	222.1479	1.3951	red
M278.098_2.7589	Positive	278.098	2.7589	red
M314.1586_1.2442	Positive	314.1586	1.2442	red
M82.0144_0.2527	Positive	82.0144	0.2527	red
M252.159_1.3688	Positive	252.159	1.3688	red
M110.0089_0.2501	Positive	110.0089	0.2501	red
M-607.3496_3.3497	Negative	607.3496	3.3497	salmon
M-593.3347_3.052	Negative	593.3347	3.052	salmon
M-333.1808_3.4906	Negative	333.1808	3.4906	salmon
M-319.1666_2.96	Negative	319.1666	2.96	salmon
M-594.3357_3.0399	Negative	594.3357	3.0399	salmon
M210.1115_1.2724	Positive	210.1115	1.2724	salmon
M388.258_2.8801	Positive	388.258	2.8801	salmon
M166.0792_0.4289	Positive	166.0792	0.4289	salmon
M321.1797_2.9397	Positive	321.1797	2.9397	salmon
M343.1615_2.9407	Positive	343.1615	2.9407	salmon
M625.3596_3.1948	Positive	625.3596	3.1948	salmon
M164.0696_2.9426	Positive	164.0696	2.9426	salmon

M-203.082_1.3884	Negative	203.082	1.3884	tan
M-429.1542_1.3846	Negative	429.1542	1.3846	tan
M-173.1027_0.2598	Negative	173.1027	0.2598	tan
M-225.0873_1.1103	Negative	225.0873	1.1103	tan
M188.0698_1.387	Positive	188.0698	1.387	tan
M146.0598_1.3879	Positive	146.0598	1.3879	tan
M175.1187_0.2681	Positive	175.1187	0.2681	tan
M159.0914_1.3869	Positive	159.0914	1.3869	tan
M104.0544_0.3797	Positive	104.0544	0.3797	tan
M132.0801_1.3844	Positive	132.0801	1.3844	tan
M205.0974_1.3799	Positive	205.0974	1.3799	tan
M468.3441_6.6781	Positive	468.3441	6.6781	tan
M118.0654_1.3784	Positive	118.0654	1.3784	tan
M-151.0392_2.576	Negative	151.0392	2.576	turquoise
M199.1296_2.8409	Positive	199.1296	2.8409	turquoise
M349.1816_1.7948	Positive	349.1816	1.7948	turquoise
M305.1555_1.6472	Positive	305.1555	1.6472	turquoise
M327.1993_1.7961	Positive	327.1993	1.7961	turquoise
M393.2072_1.9126	Positive	393.2072	1.9126	turquoise
M371.2254_1.9148	Positive	371.2254	1.9148	turquoise
M261.1299_1.4651	Positive	261.1299	1.4651	turquoise
M476.3043_2.1251	Positive	476.3043	2.1251	turquoise
M481.26_2.1275	Positive	481.26	2.1275	turquoise
M415.2507_2.0216	Positive	415.2507	2.0216	turquoise
M432.278_2.0198	Positive	432.278	2.0198	turquoise
M437.2338_2.0208	Positive	437.2338	2.0208	turquoise
M520.3307_2.2154	Positive	520.3307	2.2154	turquoise
M599.3729_3.0031	Positive	599.3729	3.0031	turquoise
M579.8357_2.9689	Positive	579.8357	2.9689	turquoise
M525.2859_2.2148	Positive	525.2859	2.2148	turquoise
M459.2783_2.1263	Positive	459.2783	2.1263	turquoise
M553.9936_3.2632	Positive	553.9936	3.2632	turquoise
M332.3148_4.5747	Positive	332.3148	4.5747	turquoise
M539.3185_3.2459	Positive	539.3185	3.2459	turquoise
M524.6431_3.2216	Positive	524.6431	3.2216	turquoise
M552.3426_3.2621	Positive	552.3426	3.2621	turquoise
M503.3035_2.2154	Positive	503.3035	2.2154	turquoise
M568.8455_2.962	Positive	568.8455	2.962	turquoise
M537.6644_3.2471	Positive	537.6644	3.2471	turquoise
M731.4499_3.1635	Positive	731.4499	3.1635	turquoise
M509.9669_3.1999	Positive	509.9669	3.1999	turquoise
M504.2921_3.1997	Positive	504.2921	3.1997	turquoise
M489.6166_3.1652	Positive	489.6166	3.1652	turquoise
M518.966_3.2279	Positive	518.966	3.2279	turquoise
M531.9926_3.2409	Positive	531.9926	3.2409	turquoise
M517.3158_3.2203	Positive	517.3158	3.2203	turquoise
M522.9945_3.2189	Positive	522.9945	3.2189	turquoise

M775.4764_3.2196	Positive	775.4764	3.2196	turquoise
M753.4643_3.1995	Positive	753.4643	3.1995	turquoise
M474.94_3.1409	Positive	474.94	3.1409	turquoise
M533.6421_3.2482	Positive	533.6421	3.2482	turquoise
M546.6664_3.2599	Positive	546.6664	3.2599	turquoise
M797.4911_3.2442	Positive	797.4911	3.2442	turquoise
M502.6408_3.2022	Positive	502.6408	3.2022	turquoise
M582.3245_2.9757	Positive	582.3245	2.9757	turquoise
M495.29_3.1667	Positive	495.29	3.1667	turquoise
M487.9664_3.1639	Positive	487.9664	3.1639	turquoise
M613.3412_3.0313	Positive	613.3412	3.0313	turquoise
M473.2912_3.1413	Positive	473.2912	3.1413	turquoise
M634.8873_3.0669	Positive	634.8873	3.0669	turquoise
M511.6344_3.221	Positive	511.6344	3.221	turquoise
M460.266_3.1097	Positive	460.266	3.1097	turquoise
M560.3264_2.9648	Positive	560.3264	2.9648	turquoise
M496.9611_3.2015	Positive	496.9611	3.2015	turquoise
M480.6151_3.1412	Positive	480.6151	3.1412	turquoise
M652.8072_2.3926	Positive	652.8072	2.3926	turquoise
M-180.0658_0.4427	Negative	180.0658	0.4427	yellow
M-130.0864_0.5395	Negative	130.0864	0.5395	yellow
M-383.1217_0.4362	Negative	383.1217	0.4362	yellow
M-154.0594_0.2714	Negative	154.0594	0.2714	yellow
M-116.071_0.3575	Negative	116.071	0.3575	yellow
M-282.0853_0.5009	Negative	282.0853	0.5009	yellow
M-283.1636_0.5378	Negative	283.1636	0.5378	yellow
M-148.043_0.3914	Negative	148.043	0.3914	yellow
M-271.0705_1.389	Negative	271.0705	1.389	yellow
M-445.1876_0.536	Negative	445.1876	0.536	yellow
M-163.039_0.4404	Negative	163.039	0.4404	yellow
M-576.2819_0.5354	Negative	576.2819	0.5354	yellow
M-222.0482_0.2684	Negative	222.0482	0.2684	yellow
M86.0958_0.5314	Positive	86.0958	0.5314	yellow
M136.0752_0.4379	Positive	136.0752	0.4379	yellow
M123.0439_0.4358	Positive	123.0439	0.4358	yellow
M165.0539_0.4362	Positive	165.0539	0.4362	yellow
M119.0489_0.4356	Positive	119.0489	0.4356	yellow
M147.0435_0.4363	Positive	147.0435	0.4363	yellow
M132.1013_0.5289	Positive	132.1013	0.5289	yellow
M182.0807_0.4362	Positive	182.0807	0.4362	yellow
M133.0316_0.3932	Positive	133.0316	0.3932	yellow
M69.07_0.4975	Positive	69.07	0.4975	yellow
M95.0489_0.4347	Positive	95.0489	0.4347	yellow
M91.0537_0.4339	Positive	91.0537	0.4339	yellow
M87.0282_0.3807	Positive	87.0282	0.3807	yellow
M136.0737_1.1909	Positive	136.0737	1.1909	yellow
M202.0857_1.9844	Positive	202.0857	1.9844	yellow

M216.0144_0.2664	Positive	216.0144	0.2664	yellow
M61.0112_0.3925	Positive	61.0112	0.3925	yellow
M235.0591_0.2686	Positive	235.0591	0.2686	yellow
M447.2007_0.5182	Positive	447.2007	0.5182	yellow

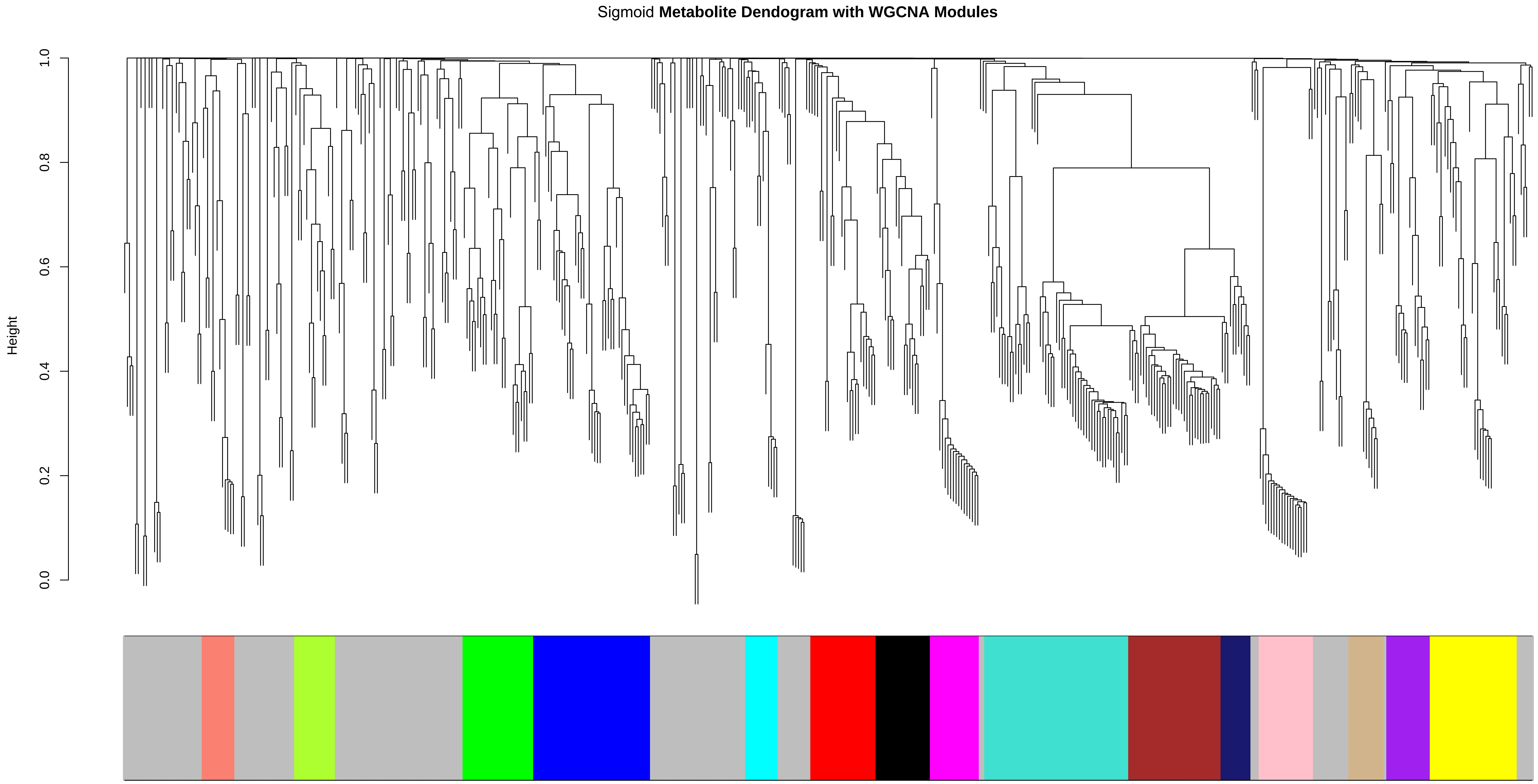
Sigmoid **Metabolite Dendogram with WGCNA Modules**
Height
0.0
0.2
0.4
0.6
0.8
1.0
