# Supplementary material for: Integrative analysis of the microbiome and metabolome of the human intestinal mucosal surface reveals exquisite inter-relationships
Source: Microbiome. 2013 Jun 5;1:17. doi: 10.1186/2049-2618-1-17 (PMC3971612; doi:10.1186/2049-2618-1-17)
Supplement: Additional file 11 — Microbial community structure potentially driven by an inhibitory metabolite. A heat map generated from a SparCC-correlated matrix of operational taxonomic units (OTUs) is shown; they were all either positively or negatively correlated with a single metabolite (mass = 434.1867, retention time = 0.6621 minutes). OTUs from the Firmicutes clade were exclusively positively associated with the metabolite, while OTUs from the Proteobacteria clade were exclusively negatively associated with the metabolite. The SparCC heat map shows these two phylogenetically distinct communities were extremely co-exclusive. The microbial IDs are shown to the left of each corresponding row in the heat map. One potential explanation for this phenomenon is if the metabolite is an inhibitory metabolite that specifically targets members of the Proteobacteria clade that is produced by OTUs from the Firmicutes clade. [file 2049-2618-1-17-S11.pdf]

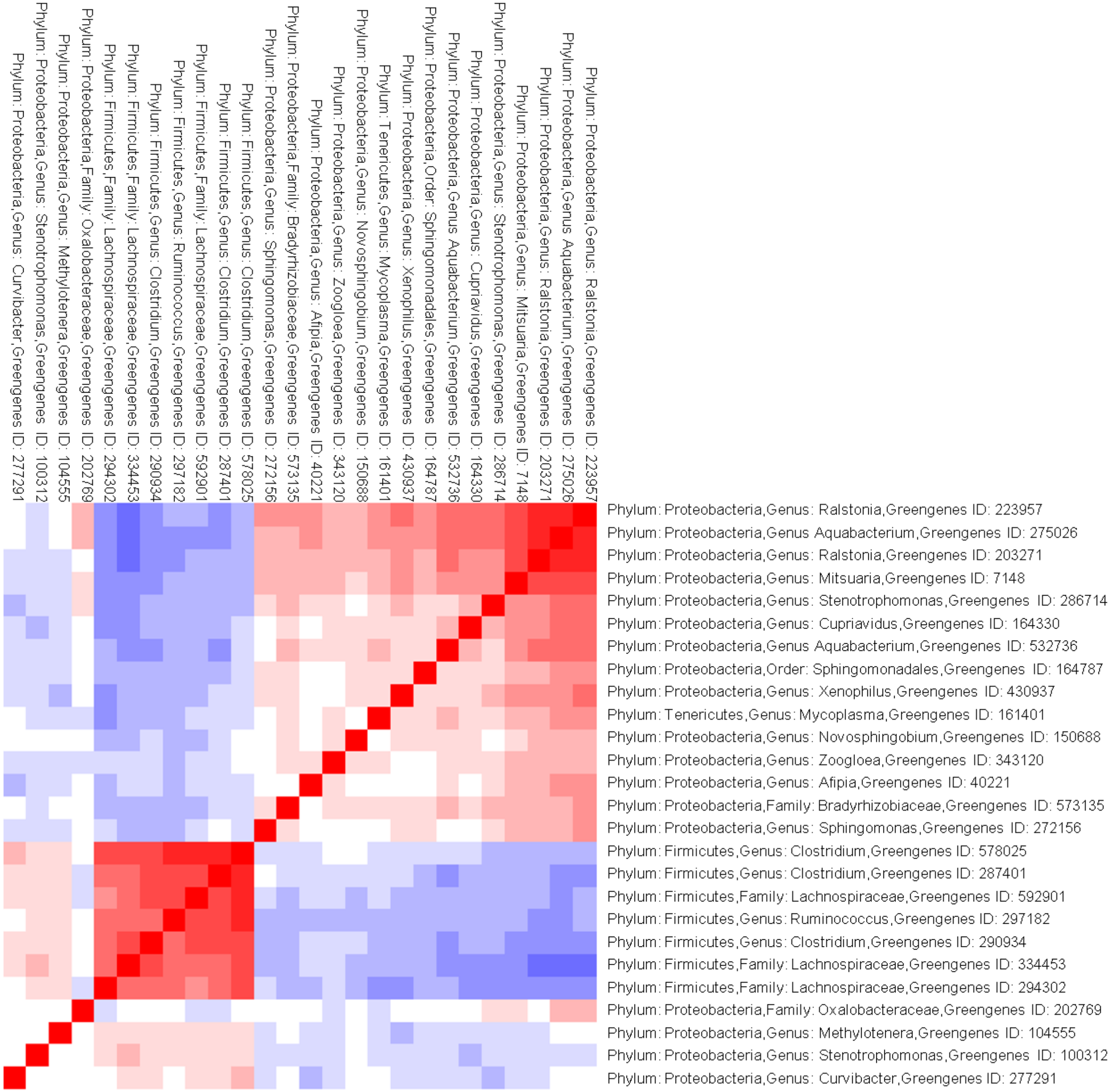
Phylum: Proteobacteria,Genus: Ralstonia,Greengenes ID: 223957
Phylum: Proteobacteria,Genus Aquabacterium,Greengenes ID: 275026
Phylum: Proteobacteria,Genus: Ralstonia,Greengenes ID: 203271
Phylum: Proteobacteria,Genus: Mitsuaria,Greengenes ID: 7148
Phylum: Proteobacteria,Genus: Stenotrophomonas,Greengenes ID: 286714
Phylum: Proteobacteria,Genus: Cupriavidus,Greengenes ID: 164330
Phylum: Proteobacteria,Genus Aquabacterium,Greengenes ID: 532736
Phylum: Proteobacteria,Order: Sphingomonadales,Greengenes ID: 164787
Phylum: Proteobacteria,Genus: Xenophilus,Greengenes ID: 430937
Phylum: Tenericutes,Genus: Mycoplasma,Greengenes ID: 161401
Phylum: Proteobacteria,Genus: Novosphingobium,Greengenes ID: 150688
Phylum: Proteobacteria,Genus: Zoogloea,Greengenes ID: 343120
Phylum: Proteobacteria,Genus: Afipia,Greengenes ID: 40221
Phylum: Proteobacteria,Family: Bradyrhizobiaceae,Greengenes ID: 573135
Phylum: Proteobacteria,Genus: Sphingomonas,Greengenes ID: 272156
Phylum: Firmicutes,Genus: Clostridium,Greengenes ID: 578025
Phylum: Firmicutes,Genus: Clostridium,Greengenes ID: 287401
Phylum: Firmicutes,Family: Lachnospiraceae,Greengenes ID: 592901
Phylum: Firmicutes,Genus: Ruminococcus,Greengenes ID: 297182
Phylum: Firmicutes,Genus: Clostridium,Greengenes ID: 290934
Phylum: Firmicutes,Family: Lachnospiraceae,Greengenes ID: 334453
Phylum: Firmicutes,Family: Lachnospiraceae,Greengenes ID: 294302
Phylum: Proteobacteria,Family: Oxalobacteraceae,Greengenes ID: 202769
Phylum: Proteobacteria,Genus: Methylotenera,Greengenes ID: 104555
Phylum: Proteobacteria,Genus: Stenotrophomonas,Greengenes ID: 100312
Phylum: Proteobacteria,Genus: Curvibacter,Greengenes ID: 277291
