# Supplementary material for: Integrative analysis of the microbiome and metabolome of the human intestinal mucosal surface reveals exquisite inter-relationships
Source: Microbiome. 2013 Jun 5;1:17. doi: 10.1186/2049-2618-1-17 (PMC3971612; doi:10.1186/2049-2618-1-17)
Supplement: Additional file 10 — Microbial community structure potentially driven by microbial competition for a metabolite. A heat map generated from a SparCC-correlated matrix of operational taxonomic units (OTUs) that were all found to significantly negatively correlate with a single metabolite (mass = 230.1845, retention time = 0.4078 minutes). The heat map depicts two strongly co-occurrent clusters of bacteria that display co-exclusive behavior. The microbial IDs are shown to the left of each corresponding row in the heat map. This behavior could be explained by competition by the two communities for the associated metabolite. [file 2049-2618-1-17-S10.pdf]

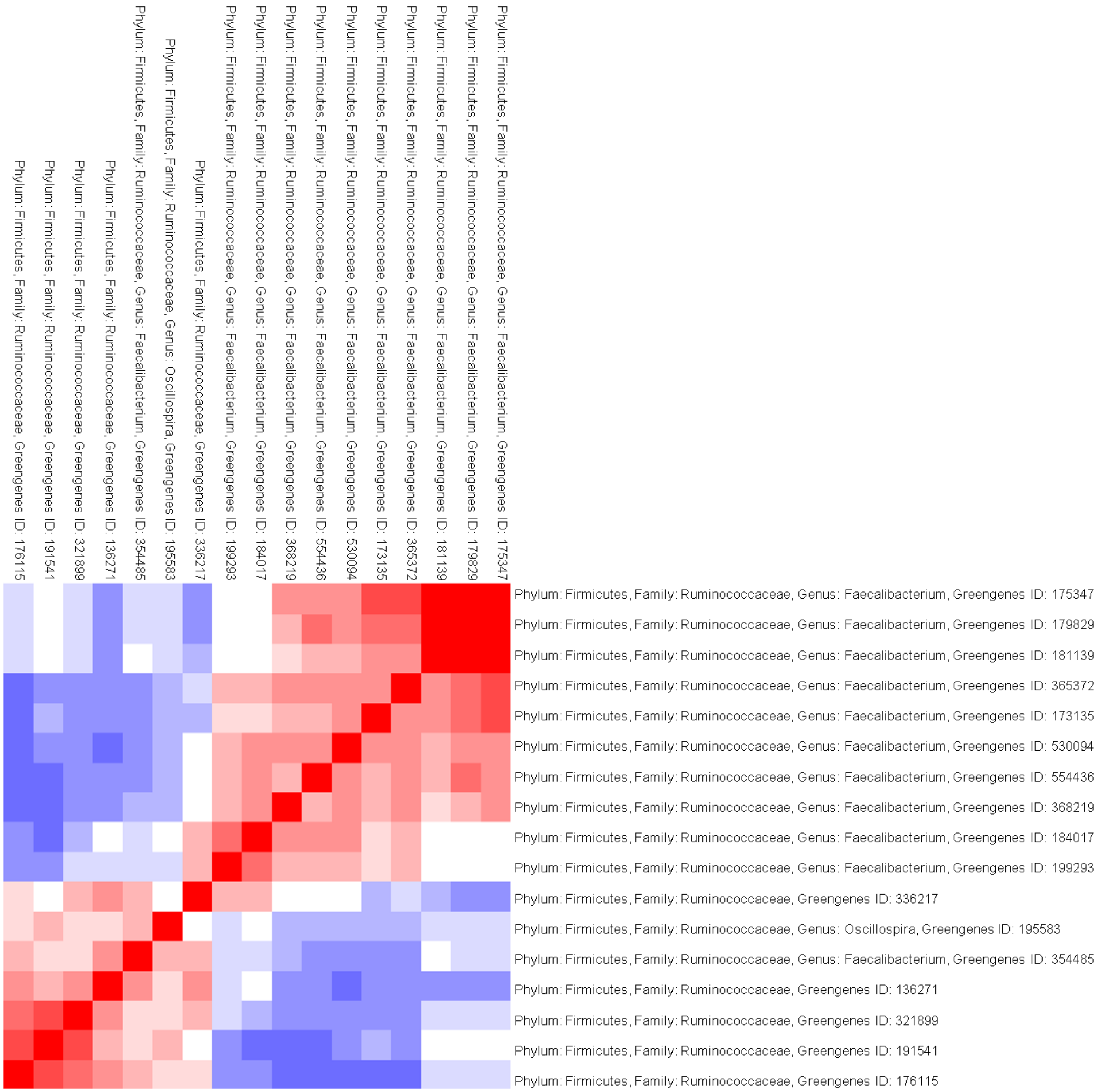

Phylum: Firmicutes, Family: Ruminococcaceae, Genus: Faecalibacterium, Greengenes ID: 175347
Phylum: Firmicutes, Family: Ruminococcaceae, Genus: Faecalibacterium, Greengenes ID: 179829
Phylum: Firmicutes, Family: Ruminococcaceae, Genus: Faecalibacterium, Greengenes ID: 181139
Phylum: Firmicutes, Family: Ruminococcaceae, Genus: Faecalibacterium, Greengenes ID: 365372
Phylum: Firmicutes, Family: Ruminococcaceae, Genus: Faecalibacterium, Greengenes ID: 173135
Phylum: Firmicutes, Family: Ruminococcaceae, Genus: Faecalibacterium, Greengenes ID: 530094
Phylum: Firmicutes, Family: Ruminococcaceae, Genus: Faecalibacterium, Greengenes ID: 554436
Phylum: Firmicutes, Family: Ruminococcaceae, Genus: Faecalibacterium, Greengenes ID: 368219
Phylum: Firmicutes, Family: Ruminococcaceae, Genus: Faecalibacterium, Greengenes ID: 184017
Phylum: Firmicutes, Family: Ruminococcaceae, Genus: Faecalibacterium, Greengenes ID: 199293
Phylum: Firmicutes, Family: Ruminococcaceae, Greengenes ID: 336217
Phylum: Firmicutes, Family: Ruminococcaceae, Genus: Oscillospira, Greengenes ID: 195583
Phylum: Firmicutes, Family: Ruminococcaceae, Genus: Faecalibacterium, Greengenes ID: 354485
Phylum: Firmicutes, Family: Ruminococcaceae, Greengenes ID: 136271
Phylum: Firmicutes, Family: Ruminococcaceae, Greengenes ID: 321899
Phylum: Firmicutes, Family: Ruminococcaceae, Greengenes ID: 191541
Phylum: Firmicutes, Family: Ruminococcaceae, Greengenes ID: 176115
